# Analysis of the Additive Effects of Nutritional Strategies in Strength Training Interventions on Body Composition, Muscle Strength and Bone Mineral Density in Postmenopausal Women: A Systematic Review

**DOI:** 10.1186/s40798-025-00954-2

**Published:** 2026-01-14

**Authors:** Franziska Walter, Jan Schalla, Wilhelm Bloch, Patrick Diel, Stephan Geisler, Eduard Isenmann

**Affiliations:** 1 Department of Molecular and Cellular Sports Medicine, Institute for Cardiovascular Research and Sports Medicine, German Sport University Cologne, 50933 Cologne, Germany; 2 Department of Fitness and Health, IST University of Applied Sciences, 40476 Duesseldorf, Germany

**Keywords:** Diet, Physical fitness, Postmenopausal women, Resistance training, Supplementation

## Abstract

**Background:**

During menopause, women experience a range of physiological changes, including reduction in skeletal muscle mass, bone mineral density, and an increase in fat mass. Although strength training and dietary strategies have individually been shown to counteract these changes, evidence for their combined effects is currently lacking. This review aims to investigate the combinatory effects on body composition, muscle strength, and bone mineral density.

**Methods:**

Three databases (PUBMED, Web of Science, and SPORTDiscus) were screened following the PRISMA guidelines. The PEDro scale was utilized to evaluate methodological quality and potential bias risk. The analyzed outcome parameters were body composition, muscle strength, and bone mineral density.

**Results:**

A total of 34 studies including postmenopausal women (*N* = 1,541) were identified; 31 of these had a PEDro score of 6 or higher. In general, body composition, muscle strength, and bone mineral density have been significantly altered through systematic strength training. Eleven studies focused on an additional calorie deficit (250-750 kcal/day) which enhanced the reduction of fat mass. Protein intake was examined in nine studies and has no significant additional effect on muscle strength and lean body mass with a minimal intake of 0.8 g/kg bodyweight. Only a few studies could be identified on other nutritional and supplementation strategies. A total of three studies were identified investigating strength training in conjunction with amino acid supplementation, four studies examining calcium and vitamin D, four studies on creatine, one study on zataria multiflora, one study on omega-3 supplementation and one study on shatavari.

**Conclusion:**

Systematic strength training has been consistently demonstrated to improve body composition, strength capacity, and bone mineral density. However, the evidence supporting the effectiveness of additional nutritional and supplementation strategies remains inconclusive. While a calorie-restricted diet and adequate protein intake appear to promote favourable changes in body composition, the available data is still insufficient to derive specific and evidence-based recommendations regarding supplementation in conjunction with strength training. Moreover, research on additional nutritional and supplementation strategies remains inconsistent or scarce, underscoring the need for further studies to allow for more precise recommendations.

*PROSPERO Registration Number* CRD42023412915 (12th April 2023).

**Supplementary Information:**

The online version contains supplementary material available at 10.1186/s40798-025-00954-2.

## Introduction

It is generally known that body composition, physical performance and functionality begin to decline steadily in the fourth decade of life [1]. In women, one of the most crucial phases in ageing is menopause, which usually occurs between the ages of 45 and 55 years [2]. Menopause is defined as 12 consecutive months of amenorrhea without surgical intervention or other causes. It marks the end of reproductive function [3]. This transition is characterized by several physiological and hormonal changes, most notably a reduction in estradiol and progesterone concentrations. These hormonal alterations contribute to a decline in skeletal muscle mass (SMM), muscle strength and bone mineral density (BMD) as well as an increase in fat mass (FM) [4–7]. It is estimated that about 3–10% of SMM is lost per decade of life after the third decade [1]. A decrease in SMM leads to reduced functionality and performance, which increases the risk of falls and fractures [8–10]. In this context, postmenopausal women with reduced SMM exhibit a 2.1-fold higher risk of falls and a 2.7-fold higher risk of fractures compared with those who have maintained SMM [11]. Overall, menopausal women are at an increased risk of developing osteoporosis and musculoskeletal disorders, including musculoskeletal pain and sarcopenic obesity [7, 12–14]. 

To maintain SMM and promote longevity, physical activity is an ideal prevention strategy to counteract the ageing process [15]. Systematic strength training (ST) in particular seems to be an ideal strategy to counteract the side effects of menopause. Recent reviews on postmenopausal women have shown that ST improves functionality, BMD [16, 17], and body composition. For example, Thomas et al. [8] found that ST achieved a small to moderate increase in lean body mass (LBM) [8]. Sá et al. [17] observed a reduction in FM when performing ST [17]. Regarding the effects of training frequency on BMD and body composition, however, the available data remains insufficient to draw definitive conclusions [16]. Concerning training duration, the meta-analysis by González-Gálvez et al [18] demonstrates that three training sessions per week with a duration of 60 min can improve strength in the lower and upper body [18]. 

In addition to physical activity, nutritional strategies are recognized crucial for promoting a healthy lifestyle. Although general dietary recommendations for postmenopausal women do not differ substantially from those for other populations [19], only a few specific guidance on nutrition and supplementation are available. A low-energy diet is recommended for postmenopausal women to counteract metabolic changes and high-fat accumulation [20]. Regarding minerals and vitamin D intake, current recommendations to preserve BMD suggest a daily calcium intake of 700 mg – 1500 mg/day and a vitamin D intake of 400–800 IU/day [21–24]. Further recommendations regarding supplementation specifically for postmenopausal women have not been established in the literature. Although evidence exists on physical activity and nutrition strategies in postmenopausal women, these two aspects are primarily considered separately. However, for a healthy lifestyle, physical activity should be combined with a balanced and nutritious diet. In younger adults, for example, studies demonstrated that the combination of ST and nutritional interventions is more effective on body composition than ST alone [25–27]. 

To date, no systematic review has examined the combined effects of ST and nutritional interventions in postmenopausal women. Accordingly, this review aims to identify and summarize the potential effects of ST in conjunction with dietary strategies. It will investigate the additive impact of nutritional interventions on body composition, muscle strength, and BMD within ST programs targeting postmenopausal women.

### Methods

### Information Source and Search

The literature search was independently completed by two researchers (JS and FW) following the PRISMA guidelines across PubMed, Web of Science, and SPORTDiscus [28]. The last search was conducted on August 5, 2025.

The study selection process, including the removal of exact duplicates, was managed using the current version of Rayyan.ai (Rayyan Systems Inc., Cambridge, USA). Any discrepancies during the selection process were resolved through consultation with a third reviewer (EI). In cases where the full text was unavailable online, the primary author and/or co-authors of the study were contacted via email by researcher FW. The systematic review was registered beforehand on PROSPERO on the April 12, 2023 (registration number: CRD42023412915).

The search strategy involved utilizing Medical Subject Headings (MeSH) and keywords, which were combined using Boolean operators (“AND”, “OR”). The used terms were: (postmenopaus*) AND ((strength*) OR (weight*) OR (resistance*) OR (barbell*) OR (machine*) OR (freeweight*)) AND ((*diet*) OR (nutrition*) OR (supple*) OR (*intake)) AND ((*performance) OR (fitness) OR (capacity) OR (muscle*) OR (lean body mass) OR (fat mass) OR (fat-free mass) OR (body composition) OR (body fat) OR (body mass) OR (BMI) OR (bone density)).

### Selection Criteria

The eligibility criteria were established using the Patient-Intervention-Comparison-Outcome-Study-Timing (PICOST) scheme. Randomized, controlled study designs (RCTs) or controlled clinical trials written in either English or German were included in the screening process (S). The aspired study population comprised healthy postmenopausal (≥ 1 year without menstruation) women (P). The intervention required a structured ST program with a reproducible exercise intensity in combination with a diet or a dietary supplementation (I). The minimum intervention duration (T) was four weeks. Diet-and-exercise intervention groups were compared with diet-only, exercise-only, non-dieting, non-exercising control, or distinct diet-and-exercise intervention groups (C). Finally, only studies evaluating relevant outcomes on health parameters for body composition such as fat-free mass (FFM), LBM, SMM or FM, muscle strength or BMD were included (O). If there were multiple reports of the same study with the same sample size, the results were treated as a single study. Exclusion criteria included studies involving animal models, studies where male participants could not be distinguished, cohorts with a mean age greater than 65 years, participants using prescribed medication, or populations with pre-existing diseases other than obesity. Studies were also excluded if the full text could not be obtained despite attempts to contact the corresponding author.

###  Data Extraction

The extracted data included information on study design, participants, performed interventions including exercises and nutritional instructions, and relevant outcome parameters. When a study compared multiple treatments or included male participants, only outcomes relevant to the research question were considered. When parameters were presented exclusively in graphical form, PlotDigitizer was used to extract mean values and standard deviations (SD) or standard errors (SE) from the figures. The standard deviation and mean value of all parameters were assessed and used to calculate the correlation coefficient r [29]. In case the SD was not provided, it was calculated by using the SE and the sample size or the confidence interval, if reported. The used formulas were as follows [29, 30]:

$$\:SD=SE\:\times\:\surd\:\left(n\right)$$

$$ r~ = ~\left( {Mdiff \div SDdiff} \right) \div \surd \left( {Mdiff \div SDdiff} \right)^{2} + 4) $$

### Quality Assessment and Risk of Bias

To evaluate the quality of all included studies the PEDro scale was used [31]. The PEDro scale includes eleven questions that address random and concealed allocation, blinding of participants, therapists and assessors, as well as quantity and quality of the outcome measures. Each question item was independently rated as (a) yes, (b) no, or (c) no information. Any discrepancies were discussed and subsequently resolved in consultation with a third researcher (EI).

## Results

###  Study Selection

**Fig. 1:**
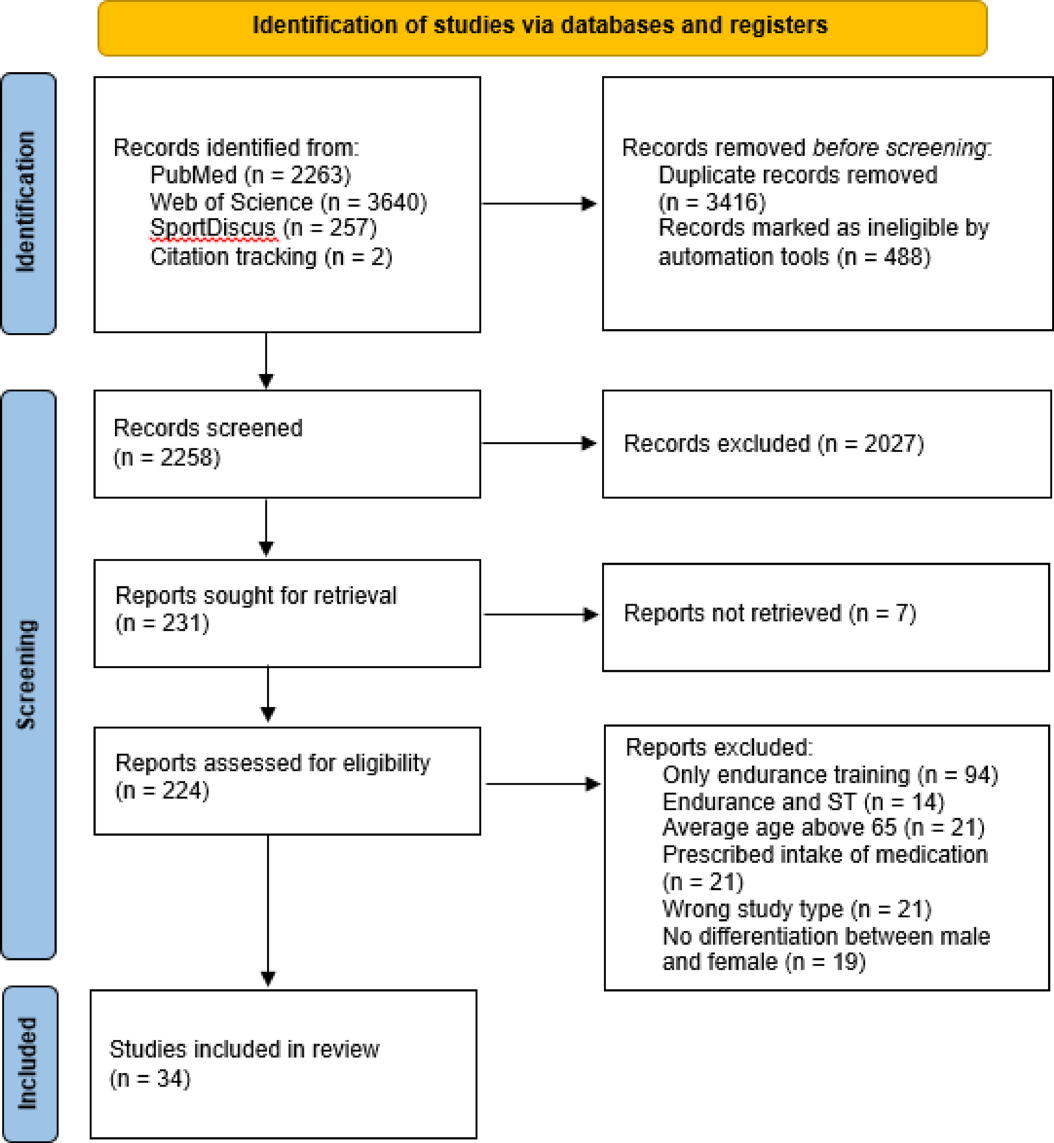
PRISMA Flow diagram [28]

Initially, a total of 7540 hits were identified, as illustrated in the PRISMA 2020 flow diagram (Fig. 1) [28]. A total of 34 studies involving 1541 postmenopausal women with an average age ranging from 53.8 to 65.0 years were included. The duration of these studies varied, ranging from four weeks to four years. The included studies that were published between 1996 and 2025. The results of the individual studies were presented according to the original data reported by the authors, either in absolute values or in relative (percentage) terms. No conversions or modifications of the data (such as rounding or reduction of decimal places) were performed. The PEDro score of the included studies ranged from poor to excellent. One study was identified as poor (score 0–3), two studies as fair (score 4–5), eighteen as good (score 6–8) and thirteen studies were identified as excellent (score 9–10). Results are shown in Table 1: PEDro Scale.

**Table 1:** PEDro scale

Study group (year)	1	2	3	4	5	6	7	8	9	10	11	Summe
Aguiar et al. (2013)	a	a	a	a	a	a	a	a	a	a	a	**10**
Bagheri et al. (2021)	a	a	a	a	a	c	a	a	a	a	a	**9**
Bouchard et al. (2009)	a	a	a	a	b	b	b	a	a	a	a	**7**
Brochu et al. (2009)	a	a	a	a	b	b	b	a	a	a	a	**7**
Candow et al. (2021)	a	a	a	a	a	a	a	b	a	a	a	**9**
Chilibeck et al. (2015)	a	a	a	a	a	a	a	b	a	a	a	**9**
Cussler et al. (2005)	a	a	c	a	b	b	b	a	a	a	a	**6**
de Branco et al. (2019)	a	a	a	a	a	a	a	a	a	a	a	**10**
Drapeau et al. (2011)	a	a	a	a	b	b	b	b	a	a	a	**6**
Félix-Soriano et al. (2021)	a	a	a	a	a	b	a	b	a	a	a	**8**
Figueroa et al. (2013) 1	a	a	a	a	b	b	b	a	a	a	a	**7**
Figueroa et al. (2013) 2	a	a	a	a	b	b	b	a	a	a	a	**7**
Figueroa et al. (2015)	a	a	a	a	a	c	c	a	a	a	a	**8**
Ghanbari-Niaki et al. (2018)	a	a	a	a	a	c	c	a	a	a	a	**8**
Greed et al. (2025)	a	a	a	a	a	a	c	c	a	a	a	**8**
Haghighi et al. (2023)	a	a	a	a	a	a	a	a	a	a	a	**10**
Holm et al. (2008)	a	a	a	b	a	a	a	b	a	a	a	**8**
Ioannidou et al. (2024)	a	a	a	a	b	b	a	a	a	a	a	**8**
Isenmann et al. (2023)	a	a	a	a	a	b	b	b	a	a	a	**7**
Johannsmeyer et al. (2016)	a	a	a	a	a	a	a	a	a	a	a	**10**
Joseph et al. (2001)	a	a	a	a	b	b	b	a	a	a	a	**7**
Kang et al. (2022)	a	a	a	a	a	c	a	a	a	a	a	**9**
Kerksick et al. (2020)	a	a	a	a	a	a	a	b	a	a	a	**9**
Maesta et al. (2007)	a	a	c	a	a	c	c	b	a	a	a	**7**
McNeil et al. (2015)	a	a	a	a	b	b	b	a	a	a	a	**7**
Molina et al. (2015)	a	c	c	c	b	b	b	c	a	a	b	**2**
Orsatti et al. (2017)	a	a	a	a	a	a	a	b	a	a	a	**9**
Rossato et al. (2017)	a	a	a	a	a	a	b	b	a	a	a	**8**
Ryan et al. (1996)	a	b	b	b	b	b	b	a	a	a	a	**4**
Ryan et al. (2003)	a	c	c	a	b	b	b	b	a	a	a	**4**
Senechal et al. (2012)	a	a	a	a	b	b	b	a	a	a	a	**7**
Shenoy et al. (2013)	a	a	a	a	b	b	b	a	a	a	a	**7**
Tayebi et al. (2019)	a	a	a	c	a	a	a	a	a	a	a	**9**
Weisgarber et al. (2015)	a	a	a	a	a	a	a	b	a	a	a	**9**

**a** *=* yes; **b** = no; **c** = no information; **1** = eligibility criteria were specified; **2** = subjects were randomly allocated to groups; **3** = allocation was concealed; **4** = the groups were similar at baseline regarding the most important prognostic indicators; **5** = there was blinding of all subjects*; **6** = there was blinding of all therapists who administered the therapy*; **7** = there was blinding of all assessors who measured at least one key outcome*; **8** = measures of at least one key outcome were obtained from more than 85% of the subjects initially allocated to groups; **9** = all subjects for whom outcome measures were available received the treatment or control condition as allocated or, where this was not the case, data for at least one key outcome was analysed by “intention to treat”; **10** = the results of between-group statistical comparisons are reported for at least one key outcome; **11** = the study provides both point measures and measures of variability for at least one key outcome; *blinding regarding nutritional strategies

### Study Designs

Six of the included studies compared a diet-only intervention with a diet-and-exercise intervention. Seventeen studies assessed the impacts of an exercise-only intervention versus a diet-and-exercise intervention. Three studies had a three-arm study design comparing exercise-only and diet-only interventions to a diet-and-exercise intervention. Data from two studies with a three-arm study design compared a diet-and-exercise intervention with a diet-only and a control intervention and three studies with a three-arm study design compared a diet-and-exercise intervention with an exercise-only and a control intervention. Eleven studies featured a four-arm study design comparing diet-and-exercise interventions with diet-only, exercise-only and control interventions, four studies compared distinct diet-and-exercise interventions, with one of them having an exercise-only control group. Finally, one study had a six-arm design with two different diet-and-exercise and two different exercise-only interventions and a diet-only and a control intervention.

### Exercise Interventions

The included studies performed ST ranging from two to four times per week. The ST was performed with resistance bands, free weights, weight machines, or in the form of whole-body vibration (WBV) training.

### Caloric Restriction Diet

Eleven studies [32–42] investigated the interaction of ST and different calorie-restrictive diets (CRD). The study periods ranged from four to twenty-six weeks. Most of the studies performed ST three times per week.

A total of five studies compared a CRD with ST to ST alone [32, 35, 36, 38, 42]. All studies showed significant differences between the groups with a significantly greater reduction in BW, body mass index (BMI), waist circumference (WC), FM and body fat (BF) percentage in the dieting groups. Besides, Bouchard et al. [32] observed a significant decline in leg extension exercises in both CRD groups (ST + CRD: 123 ± 31 kg to 108 ± 21 kg*; CRD: 104 ± 40 kg to 78 ± 33 kg*) but not in the ST group (94 ± 34 to 106 ± 29 kg) without CRD. Concerning LBM or SMM, Figueroa et al. [35, 36] detected a significant decline in the CRD groups (Figueroa et al. (2013) 1: 20.5 ± 1.0 kg to 19.5 ± 1.1 kg*; Figueroa et al. (2013) 2: 43.6 ± 1.6 kg to 42.3 to 1.8 kg*) without ST over time but not in the CRD groups with ST (Figueroa et al. (2013)1: 19.5 ± 0.8 kg to 19.1 ± 0.7 kg; Figueroa et al. (2013) 2: 42.4 ± 1.3 kg to 41.6 to 1.2 kg). However, in both studies, no significant group differences were observed. The other two studies did not show significant differences regarding LBM or muscle strength parameters between CRD groups [38, 42].

Nine studies [32–37, 39, 40, 42] compared a CRD alone versus a CRD in combination with ST. Five of these studies [33, 36, 37, 39, 40] found significant differences between the two groups. Two studies [36, 37] showed that the group combining CRD with training had a significantly greater increase in muscle strength parameters. *Figueroa et al.* demonstrated a significant increase and group effect in absolute muscle strength in the CRD + ST group (from 104 ± 5 kg to 129 ± 5 kg), compared with the CRD-only group (from 118 ± 8 kg to 111 ± 8 kg), which was similar to the effect observed in the ST-only group (113 ± 3 kg to 141 ± 8 kg) [36]. These effects are supported by observations from Joseph et al., who reported a significant difference between the groups in overall muscle strength (CRD + ST: ∆+23 ± 2 %; CRD: ∆+2 ± 2 %) [37].

Concerning FM, BF or circumferences, Bronchu et al. reported significant group differences with the combination of CRD and ST resulting in greater reductions in BF (CRD + ST: ∆ −3.7 ± 3.5 %; CRD: ∆−2.5 ± 2.7 %), trunk FM (CRD + ST: ∆−3.3 ± 2.6 kg; CRD: ∆−2.2 ± 2.5 kg), and hip circumference (HC; CRD + ST: ∆−7.5 ± 9.9 cm; CRD: ∆−4.5 ± 4.4 cm) compared with CRD alone [33]. McNeil et al. supported this findings, observing a significantly greater decline in FM in CRD + ST compared with CRD only (CRD + ST: ∆-6.1 ± 4.1 kg; CRD: ∆-3.9 ± 2.4 kg) [39]. Ryan et al. demonstrated a significant reduction in FM (33.1 ± 5.4 kg to 28.6 ± 4.6 kg) and BF (42.8 ± 3.4 % to 39.3 ± 3.7 %) following a CRD combined with ST [41]. In contrast to other studies, however, only participants with a BMI >27 kg/m² were instructed to follow the CRD, whereas the control group (BMI < 27 kg/m²) received no dietary guidance and consequently exhibited no significant changes in FM (22.2 ± 6.1 kg to 21.8 ± 6.3 kg) or BF ( 36.8 ± 5.0 % to 35.6 ± 6.1 %).

Furthermore, Joseph et al. [37] observed a significant decrease in SMM over time only in the CRD group as well as a significant between-group effect in SMM (CRD + ST: 17.9 ± 0.8 kg to 18.3 ± 0.8 kg; CRD: 16.0 ± 1.3 kg to 15.4 ± 1.3 kg). The remaining four studies [32, 34, 35, 42] did not detect any statistically significant differences between the CRD + ST and CRD only groups for body composition parameters.

With respect to BMD, only one study was identified [38]. Kerksick et al. [38] did not find any significant differences between the groups.

More detailed information is provided in Table 2.

**Table 2:** Caloric restriction

Author/ Date	Study design	Population		Training	Nutrition	Outcomes								
Bouchard et al. (2009)	Randomized controlled double-blind study	Total (*n* = 46)	obese postmenopausal	**Duration: 12 weeks**		Body composition					Muscle strength			
						BW (kg)	BMI (kg/m^2^)		FM (kg)	LBM (kg)	leg extension (lbs)		one leg squat	
		ST + CR (*n* = 12)	Ø 64.4 ± 4.5 years	**3/ week** resistance training	weight reduction by **0.5–1 kg/ week** 1 h/ week NIS **AHA diet**	77.5 ± 8.9 to 73.9 ± 9.2* r = -0.195	31.7 ± 2.6 to 30.2 ± 2.6* *r* = -0.277		33.8 ± 6.5 to 30.6 ± 6.6* r = -0.237	40.8 ± 3.2 to 40.5 ± 2.8 r = -0.050	123 ± 32 to 108 ± 21* *r* = -0.320		3.4 ± 2.6 to 6.4 ± 3.0*# *r* = 0.469	
		CR (*n* = 11)	Ø 60.7 ± 4.6 years	no supervised training		78.4 ± 10.8 to 74.4 ± 9.8* r = -0.190	31.9 ± 2.7 to 30.4 ± 2.3* *r* = -0.285		36.1 ± 7.4 to 32.9 ± 7.3* *r* = -0.213	39.2 ± 4.7 to 38.5 ± 4.1* *r* = -0.079	104 ± 40 to 78 ± 33* *r* = -0.331		2.5 ± 2.5 to 3.9 ± 4.3* *r* = 0.184	
		ST (*n* = 11)	Ø 62.8 ± 3.7 years	**3/ week** resistance training	no prescribed diet	78.5 ± 8.1 to 76.8 ± 8.2 r = -0.104	30.8 ± 2.2 to 30.7 ± 2.3 *r* = -0.022		34.4 ± 4.3 to 34.7 ± 5.1 *r* = 0.032	40.9 ± 4.3 to 41.1 ± 4.1 *r* = 0.024	94 ± 34 to 106 ± 29 *r* = 0.185		3.4 ± 1.7 to 7.0 ± 2.9*# *r* = 0.581	
		C (*n* = 12)	Ø 62.5 ± 3.1 years	no supervised training		80.1 ± 8.9 to 80.1 ± 9.4 *r* = 0	32.3 ± 2.4 to 32.6 ± 2.4 *r* = 0.062		36.9 ± 5.7 to 36.9 ± 5.8 *r* = 0	40.4 ± 4.1 to 40.1 ± 4.5 *r* = -0.035	113 ± 35 to 93 ± 35 *r* = -0.275		3.2 ± 3.0 to 3.4 ± 3.0 *r* = 0.033	
						**main outcome:** **both CR groups (CR**,** ST + CR) significantly decreased BW**,** BMI**, FM and leg extension strength over time (*) CR significantly decreased in LBM over time (*) **One leg squat increased significantly in both ST groups (ST**,** ST + CR) compared to CR and C (#)**								
Brochu et al. (2009)	Randomized double-blind, controlled study	Total (*n* = 107)	overweight/obese postmenopausal women	**Duration: 26 weeks**		body composition								
						BW (kg)	BMI (kg/m^2^)	WC (cm)	HC (cm)	BF (%)	total FM (kg)	trunk FM (kg)	total LBM (kg)	trunk LBM (kg)
		ST + CR (*n* = 36)	Ø 57.2 ± 5.0 years	**3/ week** resistance training	CR: **BRMR minus ** **500–800 kcal x 1.4 (PAF)** 2/ month NIS **AHA diet**	Baseline 84.1 ± 15.8 ∆ -5.8 ± 4.9* *r* = -0.509	Baseline 32.6 ± 4.9 ∆ -3.3 ± 1.9* *r* = -0.656	Baseline 97.1 ± 9.8 ∆ -5.6 ± 4.7* *r* = -0.512	Baseline 116.8 ± 13.1 ∆ -7.5 ± 9.9*# *r* = -0.354	Baseline 46.5 ± 4.9 ∆ -3.7 ± 3.3*# *r* = -0.489	Baseline 39.5 ± 10.5 ∆ -5.3 ± 4.3* *r* = -0.525	Baseline 19.3 ± 6.2 ∆ -3.3 ± 2.6*# *r* = -0.536	Baseline 42.1 ± 5.8 ∆ -0.4 ± 2.2* *r* = -0.091	Baseline 19.6 ± 5.1 ∆ +0.2 ± 1.6 *r* = 0.062
		CR (*n* = 71)	Ø 58.0 ± 4.7 years	no supervised training		Baseline 83.6 ± 14.4 **∆ -5.1 ± 4.7** *r* = -0.477	Baseline 32.2 ± 4.6 **∆ -1.9 ± 1.8** *r* = -0.467	Baseline 95.7 **± 9.4** ∆ -4.3 ± 3.8* *r* = -0.492	Baseline 115.9 ± 10.4 ∆ -4.5 ± 4.4* *r* = -0.455	Baseline 45.0 ± 4.3 ∆ -2.5 ± 2.7* *r* = -0.420	Baseline 37.6 ± 8.6 ∆ -4.0 ± 3.5* *r* = -0.496	Baseline 17.8 ± 4.5 ∆ -2.2 ± 2.5* *r* = -0.403	Baseline 43.0 ± 7.2 ∆ -0.9 ± 2.4* *r* = -0.184	Baseline 19.7 ± 4.0 ∆ -0.6 ± 2.0 *r* = -0.148
						**main outcome:** **The ST + CR group showed significantly greater decreases in HC**,** BF and trunk FM (#)**								
Drapeau et al. (2011)	Randomized controlled study	Total (*n* = 46)	overweight/obese postmenopausal	**Duration: 26 weeks**		body composition							no strength and bone mineral density parameters	
						BW (kg)	BMI (kg/m^2^)	BF (%)	FM (kg)	FFM (kg)	subcutaneous fat (cm^2^)	visceral fat (cm^2^)		
		ST + CR (*n* = 24)	Ø 58.0 ± 4.1 years	**3/ week** resistance training	CR: **BRMR minus ** **500–800 kcal x 1.4 (PAF)** 2/ month NIS **AHA diet**	84.4 ± 15.1 to 77.7 ± 16.6 ∆ (relative %) -8.2 ± 5.0* *r* = -0.634	32.9 ± 4.9 to 30.3 ± 5.5 ∆ (relative %) -8.1 ± 5.0* *r* = -0.629	46.6 ± 4.2 to 42.3 ± 5.9 ∆ (relative %) -9.3 ± 8.3* *r* = -0.489	39.6 ± 10.0 to 33.6 ± 12.3 ∆ (relative %) -16.5 ± 11.5* *r* = -0.583	44.6 ± 6.3 to 44.1 ± 5.4 ∆ (relative %) -0.9 ± 4.7* *r* = -0.095	465.6 ± 92.9 to 406.8 ± 101.3 ∆ (relative %) -14.6 ± 8.0* *r* = -0.674	180.1 ± 57.9 to 146.9 ± 56.5 ∆ (relative %) -19.3 ± 10.3* *r* = -0.684		
		CR (*n* = 22)	Ø 58.5 ± 4.6 years	no supervised training		82.5 ± 16.4 to 76.2 ± 15.7 ∆ (relative) -7.7 ± 3.8* *r* = -0.712	32.1 ± 5.5 to 29.7 ± 5.5 ∆ (relative) -7.7 ± 3.8* *r* = -0.712	44.3 ± 5.0 to 40.9 ± 6.6 ∆ (relative) -7.8 ± 7.9* *r* = -0.443	36.9 ± 10.0 to 31.9 ± 10.8 ∆ (relative) -14.7 ± 9.6* *r* = -0.608	45.8 ± 8.4 to 44.4 ± 7.2 ∆ (relative) -2.5 ± 5.3* *r* = -0.230	460.5 ± 122.7 to 407.1 ± 135.8 ∆ (relative) -12.6 ± 8.7* *r* = -0.587	175.8 ± 54.6 to 148.7 ± 55.3 ∆ (relative) -15.3 ± 15.3* *r* = -0.447		
						**No significant differences in between the groups**								
Figueroa et al. (2013) 1	Randomized controlled study	Total (*n* = 41)	Obese postmenopausal	**Duration: 12 weeks**		body composition						No strength parameters		
						BW (kg)	BMI (kg/m^2^)	WC (cm)	SMM (kg)	SMM index (%)				
		ST + CR (*n* = 14)	Ø 54 ± 1 years	**3/ week** low-intensity resistance training	diet: **approx. 1250 kcal/day** portion controlled foods + recommended conventional foods	86.8 ± 3.6 to 81.9 ± 3.5*# *r* = -0.181	32.7 ± 1.1 to 30.9 ± 0.9* *r* = -0.231	115 ± 3 to 110 ± 3* *r* = -0.217	19.5 ± 0.8 to 19.1 ± 0.7 *r* = -0.071	22.4 ± 0.6 to 23.4 ± 0.6* *r* = 0.217				
		CR (*n* = 14)	Ø 55 ± 1 years	no supervised training		92.2 ± 3.7 to 86.5 ± 3.6*# *r* = -0.204	35.1 ± 1.0 to 32.8 ± 1.1*# *r* = -0.280	118 ± 3 to 111 ± 3*# *r* = -0.298	20.5 ± 1.0 to 19.5 ± 1.1*# *r* = -0.126	22.3 ± 0.4 to 22.7 ± 0.6 *r* = 0.101				
		ST (*n* = 13)	Ø 54 ± 1 years	**3/ week** low-intensity resistance training	no prescribed diet	88.7 ± 4.6 to 86.5 ± 4.1 *r* = -0.070	32.6 ± 1.0 to 31.8 ± 1.1 *r* = -0.105	116 ± 4 to 115 ± 4 *r* = -0.035	20.7 ± 1.0 to 21.5 ± 1.3 *r* = 0.094	23.4 ± 0.8 to 24.3 ± 0.8 *r* = 0.154				
						**main outcome:** **the CR group had a significant decrease in BW**,** BMI**,** WC compared to ST (#)**, but also a significant decrease in SMM over time (*) the ST + CR group had a significant decrease only in BW compared to ST (#) and a significant increase in SMM index over time (*) **- no significant differences in between the CR and ST + CR group**								
Figueroa et al. (2013) 2	Randomized controlled study	Total (*n* = 41)	overweight/obese postmenopausal	**Duration: 12 weeks**		body composition							muscle strength	
						BW (kg)	total FM (kg)		trunk FM (kg)	total LM (kg)	arm LM (kg)	leg LM (kg)	Absolute (kg)	Relative (kg/kg BW)
		ST + CR (*n* = 14)	Ø 54 ± 1 years	**3/ week** low-intensity resistance training	diet: **approx. 1250 kcal/day** portioncontrolled foods + recommended conventional foods	86.7 ± 2.7 to 81.9 ± 2.5*# *r* = -0.239	41.9 ± 2.0 to 37.5 ± 2.0*# *r* = -0.282		22.1 ± 1.1 to 19.6 ± 1.2*# *r* = -0.278	42.4 ± 1.3 to 41.6 ± 1.2 *r* = -0.085	4.6 ± 0.2 to 4.5 ± 0.2 *r* = -0.067	14.9 ± 0.6 to 14.6 ± 0.5 *r* = -0.072	104 ± 5 to 129 ± 5*# *r* = 0.556	7.1 ± 0.4 to 8.8 ± 0.3* *r* = 0.533
		CR (*n* = 13)	Ø 54 ± 1 years	no supervised training		89.0 ± 4.4 to 83.4 ± 4.6*# *r* = -0.170	44.1 ± 2.9 to 39.7 ± 3.1*# *r* = -0.199		23.5 ± 1.7 to 20.8 ± 1.5*# *r* = -0.227	43.6 ± 1.6 to 42.3 ± 1.8* *r* = -0.105	4.8 ± 0.3 to 4.7 ± 0.3 *r* = -0.046	15.6 ± 0.8 to 14.6 ± 0.9*# *r* = -0.160	118 ± 8 to 111 ± 8 *r* = -0.120	7.6 ± 0.6 to 7.3 ± 0.6 *r* = -0.069
		ST (*n* = 14)	Ø 54 ± 1 years	**3/ week** low-intensity resistance training	no prescribed diet	88.4 ± 4.6 to 86.9 ± 4.3 *r* = -0.045	41.3 ± 3.2 to 40.2 ± 2.7 *r* = -0.072		20.2 ± 1.8 to 20.0 ± 1.6 *r* = -0.016	44.0 ± 2.1 to 44.2 ± 2.3 *r* = 0.012	4.6 ± 0.2 to 4.7 ± 0.3 *r* = 0.050	15.8 ± 0.9 to 16.1 ± 1.1 *r* = 0.039	113 ± 3 to 141 ± 8*# *r* = 0.471	7.3 ± 0.4 to 8.8 ± 0.8* *r* = 0.278
						**main outcome:** **The CR and the ST + CR group had a significantly greater decrease in BW**,** total and trunk FM compared to ST group (#)** **Both ST groups had a significantly greater increase in absolute**,** relative strength compared to CR group (only)(#)**								
Joseph et al. (2001)	Randomized double-blind, controlled study	Total (*n* = 19)	overweight/obese postmenopausal women	**Duration: 4 weeks**		body composition								muscle
						BW (kg)	BMI (kg/m^2^)	WC (cm)	waist to hip ratio	BF (%)	FM (kg)	FFM (kg)	SMM (kg)	total body strength (%)
		ST + CR (*n* = 10)	Ø 65 ± 2 years	**3/ week** resistance training	CR: **750 kcal less per day**	78.2 ± 2.1 to 75.0 ± 2.2* *r* = -0.229	29.4 ± 0.9 to 28.2 ± 0.9* *r* = -0.206	90.9 ± 2.1 to 88.2 ± 2.2* *r* = -0.195	0.81 ± 0.02 to 0.80 ± 0.02 *r* = -0.079	47.0 ± 1.4 to 44.6 ± 1.4* *r* = -0.262	36.9 ± 1.9 to 33.7 ± 1.9* *r* = -0.257	41.2 ± 0.6 to 41.3 ± 0.7 *r* = 0.024	17.9 ± 0.8 to 18.3 ± 0.8 *r* = 0.079	∆ +23 ± 2*# *r* = 0.866
		CR (*n* = 9)	Ø 61 ± 2 years	no supervised training		80.8 ± 2.6 to 78.8 ± 2.7* *r* = -0.185	30.4 ± 1.0 to 29.3 ± 1.0* *r* = -0.180	97.3 ± 2.7 to 93.3 ± 2.2* *r* = -0.259	0.86 ± 0.02 to 0.85 ± 0.01 *r* = -0.096	47.2 ± 1.4 to 45.2 ± 1.6* *r* = -0.216	38.3 ± 2.1 to 35.3 ± 2.2* *r* = -0.226	42.5 ± 1.3 to 42.4 ± 1.3 *r* = -0.013	16.0 ± 1.3 to 15.4 ± 1.3*# *r* = -0.077	∆ +2 ± 2 *r* = 0.164
						**main outcome:** **the ST + CR group showed a significantly greater increase in muscle strength and preserved SMM (#)**								
Kerksick et al. (2020)	Randomizedcontrolled double-blind study	Total (*n* = 128)	obese/overweight postmenopausal women (50–75 years)	**Duration: 14 weeks**		body composition				muscle strength				bone mineral density
						BW (kg)	FM (kg)	FFM (kg)	BF (%)	bench press 1RM (kg)	bench press volume (kg)	leg press 1RM (kg)	leg press volume (kg)	bone mineral content (g)
		ST + CRP (*n* = 50)		**3/ week** resistance training (30 min.)	CR (week 2–10:) **1600 kcal/day ** with high **protein** (55%)	94.6 ± 17.5 to 90.8 ± 17.0* *r* = -0.109	42.4 ± 11.0 to 38.9 ± 10.8* *r* = -0.159	46.0 ± 7.0 to 45.6 ± 6.7 *r* = -0.029	47.4 ± 4.5 to 45.4 ± 4.9* *r* = -0.208	27.9 ± 6.2 to 31.5 ± 6.9* *r* = 0.264	183 ± 98 to 174 ± 78 *r* = -0.050	139.7 ± 36.3 to 158.5 ± 34.9* *r* = 0.255	1726 ± 1034 to 1658 ± 690 *r* = -0.037	1770 ± 292 to 1775 ± 308 *r* = 0.008
		ST + CRC (*n* = 48)			CR (week 2–10:) **1600 kcal/day** with high **carbohydrates** (55%)	90.5 ± 17.3 to 85.4 ± 16.0* *r* = -0.151	39.1 ± 10.4 to 35.5 ± 10.6* *r* = -0.221	44.7 ± 7.0 to 44.2 ± 6.7 *r* = -0.036	46.3 ± 4.0 to 44.0 ± 5.1* *r* = -0.240	28.2 ± 6.1 to 30.9 ± 6.1* *r* = 0.216	166 ± 92 to 146 ± 57 *r* = -0.123	142.4 ± 32.1 to 151.5 ± 37.8* *r* = 0.128	1472 ± 854 to 1275 ± 647 *r* = -0.127	1748 ± 248 to 1731 ± 223 *r* = -0.036
		ST (*n* = 30)			no prescribed diet	84.5 ± 11.3 to 84.1 ± 12.1 *r* = -0.017	36.5 ± 8.1 to 35.9 ± 7.8 *r* = -0.094	42.0 ± 4.0 to 43.1 ± 4.6* *r* = 0.127	46.0 ± 4.7 to 44.4 ± 4.2* *r* = -0.176	26.8 ± 5.0 to 29.5 ± 4.5* *r* = 0.272	151 ± 44 to 173 ± 60 *r* = 0.200	149.8 ± 48.6 to 164.2 ± 43.7* *r* = 0.154	1530 ± 951 to 1782 ± 1192 *r* = 0.115	1681 ± 260 to 1667 ± 240 *r* = -0.028
						**main outcome:** **No significant differences between both CR groups** The CRC and the CRP group showed a significantly greater decrease in FM compared to the control (*) Control group significant increase in FFM over time (*)								
McNeil et al. (2015)	Randomized double-blind, controlled study	Total (*n* = 83)	overweight/obese postmenopausal women Ø 58.1 ± 4.8 years	**Duration: 26 weeks**		body composition				No strength and bone mineral density parameters				
						BW (kg)	FM (kg)		FFM (kg)					
		ST + CR (*n* = 28)		**3/ week** resistance training	CR: **BRMR minus ** **Ø 633 kcal x 1.4 (PAF)**	**81.5 ± 11.2 to 74.8 ± 10.9** ∆ -6.7 ± 4.5* *r* = -0.597	**37.9 ± 7.3 to 31.8 ± 8.1** ∆ -6.1 ± 4.1*# *r* = -0.597		**43.6 ± 5.5 to 43.0 ± 4.2** ∆ -0.5 ± 2.3* *r* = -0.108					
		CR (*n* = 65)		no supervised training		**83.5 ± 14.5 to 78.6 ± 18.3** ∆ -4.8 ± 6.7* *r* = -0.463	**37.8 ± 8.8 to 33.9 ± 9.2** ∆ -3.9 ± 2.4* *r* = -0.476		**45.6 ± 7.3 to 44.7 ± 6.2** ∆ -0.9 ± 2.4* *r* = -0.184					
						**main outcome:** **the ST + CR training group showed a significant greater decrease in FM than CR group**								
Ryan et al. (1996)	non-randomized	Total (*n* = 13)	overweight/obese postmenopausal	**Duration: 16 weeks**		body composition					muscle strength			
						BW (kg)	BMI (kg/m^2^)	FM (kg)	FFM (kg)	BF (%)	biceps flexion (Nm)	triceps extension (Nm)	leg extension (Nm)	leg flexion (Nm)
		ST + CR (*n* = 6)	Ø 58 ± 2 years **BMI > 27 kg/m2**	**3/ week** resistance training	weight reduction **by approx. 0.25–0.5 kg/week** 1/week NIS **AHA diet**	78.5 ± 6.1 to 74.3 ± 5.8* *r* = -0.330	30.3 ± 3.1 to 28.7 ± 2.9 *r* = 0.391	33.1 ± 5.4 to 28.6 ± 4.6* *r* = -0.409	43.3 ± 1.0 to 44.2 ± 1.5 *r* = 0.200	42.8 ± 3.4 to 39.3 ± 3.7* *r* = -0.442	25 ± 10 to 36 ± 15* *r* = 0.410	33 ± 7 to 52 ± 10* *r* = 0.540	63 ± 24 to 95 ± 22* *r* = 0.565	40 ± 15 to 64 ± 15* *r* = 0.632
		ST (*n* = 7)	Ø 58 ± 2 years **BMI < 27 kg/m2**		**AHA diet**	61.8# ± 7.9 to 61.9# ± 7.9 *r* = 0.006	23.2 ± 2.4# to 23.2 ± 2.4 *r* = 0.0	22.2 ± 6.1# to 21.8 ± 6.3 *r* = -0.040	37.6 ± 2.4# to 38.4 ± 3.2# *r* = 0.142	36.8 ± 5.0# to 35.6 ± 6.1 *r* = -0.107	18 ± 11 to 34 ± 13* *r* = 0.558	34 ± 11 to 44 ± 11* *r* = 0.273	63 ± 8 to 86 ± 21* *r* = 0.528	34 ± 11 to 57 ± 11* *r* = 0.736
						**main outcome:** **The ST + CR group showed a significant decrease in BW**,** BMI**, FM and BF over time (*) **- no significant differences in strength parameters**								
Ryan et al. (2003)	Randomized double-blind, controlled study	Total (*n* = 24)	overweight/obese postmenopausal	**Duration: 26 weeks**		body composition							no muscle strength and bone mineral density parameter	
						BW (kg)	BMI (kg/m^2)^	WC (cm)	waist to hip ratio	FM (kg)	FFM (kg)	BF (%)		
		ST + CR (*n* = 9)	Ø 57 ± 2 years	**3/ week** resistance training	CR: **250–350 kcal less per day** **AHA diet**	87.1 ± 13.9 to 81.4 ± 12.6* *r* = -0.210	32.1 ± 4.4 to 30.1 ± 4.4* *r* = -0.112	93.7 ± 10.1 to 90.4 ± 10.4* *r* = -0.161	0.81 ± 0.1 to 0.81 ± 0.1 *r* = 0.0	40.7 ± 9.5 to 35.6 ± 8.5* *r* = -0.268	45.5 ± 6.3 to 44.4 ± 5.7* *r* = -0.075	46.9 ± 5.1 to 43.9 ± 5.1* *r* = -0.284		
		CR (*n* = 15)	Ø 56 ± 1 years	no supervised training										
						**main outcome:** **no significant differences between the groups for body composition and data were not shown separately**								
Senechal et al. (2012)	Randomizedcontrolled double-blind study	Total (*n* = 38)	obese postmenopausal women Ø 62.6 ± 4.1 years (54–74 years)	**Duration: 12 weeks**		body composition						muscle strength		
						BW (kg)	WC (cm)	BF (%)	total FM (kg)	trunk FM (kg)	LBM (kg)	isometric leg strength (Nm)		
		ST + CR (*n* = 9)		**3/ week** resistance training	weight reduction **by 0.5–1 kg/ week** 1 h/ week NIS **AHA diet**	**Pre 77.5 ± 7.7** ∆ -3.8 ± 1.8*# *r* = -0.726	**Pre 96.6 ± 7.9** ∆ -5.3 ± 5.7* *r* = -0.422	**Pre 44.4 ± 4.1** ∆ -2.2 ± 1.8*# *r* = -0.521	**Pre 32.5 ± 5.9** ∆ -3.1 ± 1.8*# *r* = -0.653	Pre 16.9 ± 2.7 ∆ -2.0 ± 1.3*# *r* = -0.610	Pre 40.1 ± 2.7 ∆ -0.4 ± 0.9 *r* = -0.217	Pre 114.4 ± 20.2 ∆ -11.2 ± 17.9 *r* = -0.299		
		CR (*n* = 9)		no supervised training		**Pre 78.7 ± 11.7** ∆ -4.3 ± 2.7*# *r* = -0.623	**Pre 99.1 ± 7.2** ∆ -6.1 ± 3.9* *r* = -0.616	**Pre 47.8 ± 2.3** ∆ -1.8 ± 1.3*# *r* = -0.569	**Pre 36.2 ± 6.7** ∆ -3.2 ± 2.0*# *r* = -0.625	Pre 17.3 ± 2.4 ∆ -1.3 ± 1.1* *r* = -0.509	Pre 39.3 ± 5.2 ∆ -0.8 ± 1.2 *r* = -0.316	Pre 82.4 ± 23.6 ∆ -17.6 ± 24.9 *r* = -0.333		
		ST (*n* = 10)		**3/ week** resistance training	no prescribed diet	Pre 77.5 ± 7.7 ∆ 0.5 ± 1.7# *r* = 0.145	Pre 99.8 ± 7.1 ∆ -0.7 ± 4.9 *r* = -0.071	Pre 45.4 ± 2.4 ∆ -0.6 ± 1.5 *r* = -0.196	Pre 33.8 ± 4.2 ∆ -0.2 ± 1.4 *r* = -0.071	Pre 17.3 ± 3.5 ∆ +0.2 ± 1.6 *r* = 0.062	Pre 40.6 ± 4.0 ∆ +0.5 ± 1.0 *r* = 0.243	Pre 82.3 ± 19.8 ∆ +19.8. ± 37.5 *r* = 0.255		
		C (*n* = 10)		no supervised training		Pre 77.5 ± 7.2 ∆ -0.7 ± 1.4 *r* = -0.243	Pre 99.5 ± 6.4 ∆ -1.7 ± 0.3 *r* = -0.094	Pre 47.5 ± 3.5 ∆ -0.1 ± 1.3 *r* = -0.038	Pre 35.6 ± 5.3 ∆ -0.1 ± 1.2 *r* = -0.042	Pre 18.0 ± 3.1 ∆ -0.2 ± 1.3 *r* = -0.077	Pre 39.0 ± 3.1 ∆ -0.1 ± 1.0 *r* = -0.050	Pre 102.7 ± 26.2 ∆ -13.6 ± 38.4 *r* = -0.174		
						**main outcome:** **the CR group and ST + CR showed a significantly greater decline in BW**,** BF and total FM compared to control group (#)** **the CR group showed a significantly greater decline in BW and total FM (#)** **and the ST + CR group in BF%**,** total and trunk FM compared to ST group (#)** **- no significant difference between CR and ST + CR**								

### High-protein Diet and Protein Supplementation

Nine of the included studies [43–51] examined the effect of protein supplementation in combination with ST. Various protein sources and strategies were identified, including whey protein (10 to 40 g/serving) on training days [43–45, 50], daily soy protein supplementation (25 to 40 g/ serving) [46, 47, 49] and a general high-protein diet ranging from 1.2 to 1.8 g/kg bodyweight (kgBW) [48, 51]. The duration of the studies ranged from 8 to 24 weeks. All studies implemented ST with two, three or four sessions per week. When reported, the control groups also consumed 0.7–0.9 g of protein/kgBW.

Orsatti et al. [47] was the only study to report a significant between-group difference the groups group receiving daily soy protein supplementation (P + ST) and the ST group receiving placebo. The P + ST group showed significantly greater increase in bench press (P + ST: ∆+12.2 kg [8.5–15.9]; ST: ∆+6.9 kg [4.5–9.4]), knee extension (P + ST: ∆+6.5 kg [4.7–8.2]; ST: ∆+3.9 kg [2.2–5.5]) and total load (P + ST: ∆+23.6 kg [19.0-28.7]; ST: ∆+15.7 kg [11.1–20.4]) compared with the placebo group. Nevertheless, both groups exhibited significant improvements in all strength parameters, with high effect sizes, over the 16-week intervention. Holm et al. [44] reported a significant increase over time in LBM (42.3 ± 4.8 kg to 43.1 ± 4.8 kg) and quadriceps strength (∆+14 ± 4%) in P + ST, although no significant between-group differences were observed (ST: LBM: 42.9 ± 3.3 kg to 43.3 ± 3.3 kg; quadriceps strength: ∆+8 ± 4%). All the other studies did not detect any significant group differences between the protein-supplemented and the training controls for any outcome parameters.

Detailed information is provided in Table 3.

**Table 3:** Protein

Author/ Date	Category	Study design	Population		Training	Nutrition	Outcomes										
de Branco et al. (2019)	Protein timing	Randomized parallel group, double blind study	Total (*n* = 34)	Postmenopausal Ø 60.9 ± 6.7 years	**Duration: 8 weeks**		Body composition							Muscle strength			
							BW (kg)	BMI (kg/m^2^)	SMM index (kg/m^2^)	total LM (kg)	Total FM (kg)			Bench press (kg)	leg extension (kg)	Handgrip strength (kg)	
			ST + P immediately (*n* = 17)	Ø 60.5 years (57.7–63.3)	**3/ week** resist. training	**immediately** after exercise: 30 g of whey protein after 6 h: 30 g of carbohydrate	69.3 [62.6–76.6] to 69.6 [62.7–77.3] *r* = 0.011	27.9 [25.2–30.8] to 28.1 [25.4–31.1]* *r* = 0.018	6.9 [6.4–7.5] to 7.1 [6.6–7.6]* *r* = 0.098	37.3 [35.0-39.7] to 38.1 [35.9–40.5]* *r* = 0.088	29.4 [24.5–35.4] to 29.3 [24.4–35.3] *r* = -0.005			29.2 [27.1–31.3] to 33.8 [31.4–36.6]* *r* = 0.444	30.0 [26.5–33.8] to 33.8 [31.0–37.0]* *r* = 0.278	27.0 [25.6–28.5] to 28.3 [26.5–30.2]* *r* = 0.195	
			ST + P 6 h post (*n* = 17)	Ø 61.4 years (58.0–65.0)		immediately after exercise: 30 g of carbohydrate **after 6 h**: 30 g of whey protein	70.9 [65.6–76.7] to 71.8 [66.2–77.9] *r* = 0.058	27.6 [25.7–29.7] to 27.8 [25.8–29.9]* *r* = 0.026	6.8 [6.4–7.3] to 7.0 [6.6–7.5]* *r* = 0.114	38.2 [36.0-40.5] to 38.8 [36.5–41.3]* *r* = 0.066	30.6 [27.2–34.5] to 30.5 [27.0-34.3] *r* = -0.007			29.3 [26.2–32.7] to 34.8 [31.3–38.8]* *r* = 0.372	30.7 [26.5–35.7] to 33.3 [29.4–37.8] *r* = 0.150	27.4 [24.8–30.2] to 29.3 [26.6–32.1] *r* = 0.176	
							**Main outcome**: **no significant differences between groups**										
Holm et al. (2008)	Protein supplement	Randomizedcontrolled double-blind study	Total (*n* = 29)	postmenopausal women	**Duration: 24 weeks**		body composition							muscle	bone mineral density		
							SMM (quadriceps)			FM (kg)	LBM (kg)			quadriceps strength (%)	total body (g/mm^3^)	Femoral (g/mm^3^)	Lumbar (g/mm^3^)
							Proximal (mm^2^)	Mid (mm^2^)	Distal (mm^2^)								
			ST + P (*n* = 13)	Ø 55 ± 1 years	**3/ week** resist. training	after exercise: 10 g of protein (whey protein), 31 g of carbohydrate, 1 g of fat, 5 g of vitamin D and 250 mg of calcium	2894 ± 108 to 3071 ± 124* *r* = 0.218	5099 ± 103 to 5386 ± 124* *r* = 0.346	4307 ± 189 to 4622 ± 202* *r* = 0.144	21.1 ± 4.7 to 20.6 ± 4.7 *r* = -0.051	42.3 ± 4.8 to 43.1 ± 4.8* *r* = 0.079			∆ +14 ± 4* *r* = 0.437	1.113 ± 0.027 to 1.116 ± 0.027 *r* = 0.015	0.953 ± 0.051 to 0.978 ± 0.043 *r* = 0.073	1.084 ± 0.053 to 1.108 ± 0.049* *r* = 0.065
			ST (*n* = 16)	Ø 55 ± 1 years		after exercise: 6 g of carbohydrate and 12 mg of calcium	3380 ± 121 to 3566 ± 140* *r* = 0.143	5565 ± 197 to 5838 ± 196* *r* = 0.431	4442 ± 158 to 4630 ± 166* *r* = 0.174	26.5 ± 10.2 to 25.8 ± 10.2 *r* = -0.033	42.9.1 ± 3.3 to 43.3 ± 3.3 *r* = 0.065			∆ +8 ± 4 *r* = 0.267	1.117 ± 0.022 to 1.122 ± 0.023 *r* = 0.028	0.943 ± 0.028 to 0.930 ± 0.024 *r* = -0.062	1.043 ± 0.032 to 1.068 ± 0.038* *r* = 0.088
							**Main outcome:** **no significant differences between the groups**										
Isenmann et al. (2023)	Protein supplement	Randomized single-blind study	Total (*n* = 24)	postmenopausal women	**Duration: 10 weeks**		body composition							muscle strength			
							BW (kg)	BMI (kg/m^2^)	FFM (kg)	SMM (kg)	FM (kg)			left handgrip (kg)	right handgripp (kg)	squat 1RM (kg)	bench press 1RM (kg)
			mST + P (*n* = 10)	Ø 54.3 ± 4.7 years	**2/ week** moderate-intensity resistance training	after exercise: carbohydrate/ protein rich meal (one of three options, 30.7–38.4 g protein and 45.5–50.9 g carbohydrates)	70.4 ± 8.4 to 70.0 ± 8.3 *r* = -0.023	25.5 ± 2.5 to 25.4 ± 2.3 *r* = -0.032	48.1 ± 3.9 to 47.0 ± 4.4 *r* = -0.128	27.8 ± 2.2 to 27.6 ± 2.7 *r* = -0.037	22.8 ± 7.3 to 23.1 ± 6.1 *r* = -0.019			31.5 ± 3.8 to 31.0 ± 3.5 *r* = -0.077	33.3 ± 3.6 to 33.1 ± 4.5 *r* = -0.026	36.8 ± 21.5 to 60.7 ± 18.9* *r* = 0.507	27.1 ± 2.9 to 33.1 ± 5.5* *r* = 0.539
			lST + P (*n* = 12)	Ø 55.6 ± 2.9 years	**2/ week** low-intensity resistance training		69.7 ± 7.9 to 69.6 ± 8.3 *r* = -0.007	25.1 ± 2.9 to 25.1 ± 3.1 *r* = 0.010	49.2 ± 4.6 to 48.5 ± 4.0 *r* = -0.085	29.8 ± 3.2 to 28.8 ± 2.1 *r* = -0.178	20.5 ± 3.9 to 21.3 ± 4.9 *r* = 0.094			27.2 ± 5.1 to 28.7 ± 4.4 *r* = 0.159	29.8 ± 5.9 to 29.8 ± 4.7 *r* = -0.004	35.5 ± 9.2 to 62.7 ± 15.9* *r* = 0.703	27.5 ± 3.2 to 33.4 ± 3.6* *r* = 0.654
							**Main outcome**: **no significant differences between the groups**										
Ioannidou et al. (2024)	High-Protein Diet	Randomized Controlled Trial	Total (*n* = 55)	postmenopausal (58.2 ± 5.6 years)	**Duration: 12 weeks**		body composition				Muscle strength					No bone mineral density parameter	
							BW (kg)	FFM (kg)	SMM (kg)	FM (kg)	Hand grip strength (kg)			squat 1RM (kg)	deadlift 1RM (kg)		
			ST + P (*n* = 15)	Ø 57.0 ± 6.1 years	**3/ week** resistance training	**1.7 g/kgBW/day**	71.6 ± 8.9 to 72.3 ± 9.1 ∆ +1.3 ± 6.7 *r* = 0.040	**47.3 ± 5.5 to 48.6 ± 5.9** ∆ +1.3 ± 1.6* *r* = 0.110	**19.8 ± 3.3 to 21.2 ± 3.5** ∆ +1.4 ± 0.9*# *r* = 0.200	**25.9 ± 8.5 to 24.4 ± 8.4** ∆ -1.5 ± 2.0*# *r* = -0.090	**29.5 ± 2.8 to 34.2 ± 3.3** ∆ +4.7 ± 2.4*# *r* = 0.610			**47.5 ± 12.1 to 77.5 ± 20.3** ∆ +30.0 ± 14.2*# *r* = 0.650	**56.4 ± 13.0 to 77.2 ± 13.3** ∆ +20.8 ± 10.3*# *r* = 0.620		
			ST (*n* = 12)	Ø 57.6 ± 5.1 years	**3/ week** resistance training	no specific introduction **0.9 g/kgBW/day**	68.1 ± 7.6 to 68.4 ± 8.1 ∆ +0.3 ± 0.9 *r* = 0.02	49.9 ± 8.1 to 50.6 ± 8.3 ∆ +0.6 ± 2.6 *r* = 0.04	**20.6 ± 3.4 to 21.8 ± 3.8** ∆ +1.2 ± 1.3*# *r* = 0.160	**23.2 ± 8.1 to 20.8 ± 7.4** ∆ -2.4 ± 2.9*# *r* = -0.15	**29.8 ± 3.8 to 33.4 ± 3.6** ∆ +3.6 ± 3.0*# *r* = 0.39			**42.7 ± 12.1 to 76.7 ± 12.1** ∆ +34.0 ± 12.0*# *r* = 0.81	**48.3 ± 10.3 to 70.4 ± 8.4** ∆ +22.1 ± 7.6*# *r* = 0.76		
			P (*n* = 14)	Ø 56.8 ± 5.4 years	no specific intervention	**1.8 g/kgBW/day**	65.3 ± 7.7 to 66.5 ± 8.2 ∆ +1.2 ± 2.4 *r* = 0.080	46.2 ± 3.3 to 46.5 ± 2.8 ∆ +0.3 ± 2.2 *r* = 0.050	20.1 ± 1.7 to 20.1 ± 1.5 ∆ +0.0 ± 1.5 *r* = 0.000	18.9 ± 5.7 to 19.9 ± 6.0 ∆ +0.9 ± 1.8 *r* = 0.088	**28.4 ± 2.8 to 30.0 ± 3.3** ∆ +1.7 ± 3.9* *r* = 0.249			**57.2 ± 14.5 to 64.7 ± 17.1** ∆ +7.5 ± 5.4*# *r* = 0.230	**54.9 ± 10.7 to 60.5 ± 11.0** ∆ +5.5 ± 7.7*# *r* = 0.25		
			C (*n* = 14)	Ø 60.7 ± 4.8 years	no specific intervention	no specific intervention **0.7 g/kgBW/day**	73.0 ± 11.5 to 72.4 ± 11.4 ∆ -0.6 ± 1.4 *r* = -0.029	44.6 ± 7.9 to 43.8 ± 7.6 ∆ -0.8 ± 1.5 *r* = -0.049	19.6 ± 3.3 to 19.2 ± 3.5 ∆ -0.4 ± 1.0 *r* = -0.06	29.1 ± 9.9 to 29.2 ± 9.4 ∆ +0.2 ± 1.7 *r* = 0.009	29.2 ± 5.4 to 28.0 ± 6.5 ∆ -1.1 ± 2.2 *r* = -0.007			40.8 ± 16.5 to 40.4 ± 14.1 ∆ -0.4 ± 3.85 *r* = -0.008	44.8 ± 13.6 to 45.4 ± 13.4 ∆ +0.5 ± 3.9 *r* = 0.019		
							**main outcome:** **-in comparison to the non-exercising control groups (P**,** C):** **The training groups (ST + P**,** ST) showed significant increases in all strength parameters and in SMM (#) and a significant reduction in FM (#)** **- in comparison to the control group (C): ** **The diet group (P) showed a significant increase in squat and deadlift strength (#)** **No significant difference between the training groups (ST + P**,** ST)**										
Maesta et al. (2007)	Daily Soy Protein	Randomized controlled double-blind study	Total (*n* = 46)	postmenopausal	**Duration: 16 weeks**		body composition				No strength and bone mineral density parameters						
							BMI (kg/m^2^)	WC (cm)	SMM (kg)	BF (%)							
			ST + P (*n* = 14)	Ø 57.6 ± 6.7 years	**3/ week** resistance training	**Daily**: 25 g of soy protein (containing 50 mg of isoflavones) form: powder to be incorporated into 200mL of skimmed milk	27.8 ± 4.0 to 28.1 ± 4.0 ∆ +0.3 ± 0.5 *r* = 0.287	**90.9 ± 9.1 to 89.6 ± 10.1** ∆ -1.3 ± 0.9* *r* = -0.585	**19.7 ± 3.3 to 21.0 ± 3.6** ∆ +1.3 ± 0.9* *r* = 0.585	32.2 ± 6.3 to 31.9 ± 6.8 ∆ -0.3 ± 4.4 *r* = -0.034							
			P (*n* = 10)	Ø 61.3 ± 5.3 years	no supervised training		27.2 ± 5.3 to 26.9 ± 5.1 ∆ -0.3 ± 0.7 *r* = -0.210	91.8 ± 14.4 to 91.3 ± 14.4 ∆ -0.6 ± 1.3 *r* = -0.225	18.3 ± 2.4 to 18.8 ± 2.5 ∆ +0.5 ± 1.0 *r* = 0.243	30.8 ± 7.0 to 29.1 ± 4.0 ∆ -1.8 ± 3.6 *r* = -0.243							
			ST (*n* = 11)	Ø 60.7 ± 7.1 years	**3/ week** resistance training	**Daily**: 25 g of maltodextrine form: powder to be incorporated into 200mL of skimmed milk	27.7 ± 4.4 to 28.3 ± 4.9 ∆ 0.6 ± 1.3 *r* = 0.225	**90.5 ± 9.8 to 88.3 ± 10.2** ∆ -2.1 ± 2.0* *r* = -0.465	**17.8 ± 2.5 to 19.2 ± 2.8** ∆ +1.3 ± 1.2* *r* = 0.476	33.3 ± 8.3 to 32.9 ± 8.7 ∆ 0-0.3 ± 0.8 *r* = -0.184							
			C (*n* = 11)	Ø 57.9 ± 6.9 years	no supervised training		26.6 ± 3.6 to 26.6 ± 3.8 ∆ 0.0 ± 0.5 *r* = -0.030	92.0 ± 7.9 to 91.6 ± 9.3 ∆ -0.4 ± 3.1 *r* = -0.064	17.8 ± 2.7 to 18.6 ± 2.9 ∆ +0.7 ± 1.2 *r* = 0.280	31.7 ± 5.9 to 30.3 ± 5.7 ∆ -1.4 ± 3.5 *r* = -0.196							
							**No significant differences between the two training groups**										
Orsatti et al. (2017)	Daily Soy Protein	Randomized controlled study	Total (*n* = 32)	postmenopausal women	**Duration: 16 weeks**		Body composition		Muscle strength						No bone mineral density		
							SMM		bench press	lat. pull down	Knee extension			total loads			
			ST + P (*n* = 16)	Ø 56.8 ± 6.6 years	**3/ week** resistance training	**Daily**: 25 g of soy protein (containing 50 mg of isoflavones) form: powder to be incorporated into 200mL of skimmed milk	19.5 ± 3.1 to 20.7 ± 3.5 ∆ +1.2 [0.7–1.7] *r* = 0.539		**33.4 ± 7.1 to 45.5 ± 8.8** ∆ +12.2 [8.5–15.9]*# *r* = 0.669	30.3 ± 6.7 to 35.7 ± 5.5 ∆ 5.7[4.1–7.4]* *r* = 0.677	**28.1 ± 6.8 to 34.7 ± 6.7** ∆ +6.5 [4.7–8.2]*# *r* = 0.703			**91.8 ± 16.1 to 115.5 ± 15.7** ∆ +23.6 [19.0-28.7]*# *r* = 0.814			
			ST (*n* = 16)	Ø 58.8 ± 8.9 years		**Daily**: 25 g of maltodextrin form: powder to be incorporated into 200mL of skimmed milk	18.0 ± 2.4 to 19.5 ± 2.7 ∆ +1.5 [1.0-1.9] *r* = 0.624		**29.1 ± 7.6 to 36.0 ± 8.5** ∆ +6.9 [4.5–9.4]* *r* = 0.601	**27.6 ± 6.3 to 32.0 ± 6.7** ∆ +4.4 [2.7–6.2]* *r* = 0.557	**26.0 ± 5.3 to 29.6 ± 5.2** ∆ +3.9 [2.2–5.5]* *r* = 0.534			**82.6 ± 17.5 to 96.8 ± 19.3** ∆ +15.7 [11.1–20.4]* *r* = 0.669			
							**Main outcome:** **- in comparison to the ST group:** **The ST + P group showed a significant increase in three of the four strength parameters compared to control (being bench press**,** knee extension and total of loads) (#)**										
Rossato et al. (2017)	High-Protein Diet	Randomized parallel-group, single-blind study	Total (*n* = 23)	Postmenopausal women	**Duration: 10 weeks**		body composition							muscle strength			
							Total LM (kg)		Leg LM (kg)	Trunk LM (kg)	Total FM (kg)			Leg extension		Bench press	
			ST + high P (*n* = 12)	Ø 63.4 ± 7.6 years	**3/ week** resistance training	**1.2 g protein /kg/day** with both groups instructed to ingest similar amounts of protein after training (20–30 g)	**37.1 ± 6.2 to 38.4 ± 6.5** ∆ +1.3 ± 0.7* *r* = 0.259		**12.7 ± 2.9 to 13.2 ± 3.2** ∆ +0.5 ± 0.5* *r* = 0.063	15.8 ± 7.2 to 15.7 ± 7.2 ∆ 0.0 ± 0.7 *r* = 0.000	28.6 ± 11.6 to 28.3 ± 11.4 ∆ -0.3 ± 1.1 *r* = 0.135			75.3 ± 14.8 to 84.7 ± 14.6* *r* = 0.137		32.2 ± 6.3 to 34.3 ± 7.9* *r* = 0.383	
			ST + low P (*n* = 11)	Ø 63.0 ± 8.6 years		**0.8 g protein /kg/day** with both groups instructed to ingest similar amounts of protein after training (20–30 g)	**37.6 ± 6.2 to 38.8 ± 6.5** ∆ +1.3 ± 0.8* *r* = 0.210		**12.7 ± 2.6 to 13.1 ± 2.7** ∆ 0.4 ± 0.8* *r* = 0.110	15.3 ± 5.3 to 15.2 ± 0.5 ∆ -0.2 ± 0.8 *r* = 0.124	28.0 ± 9.4 to 27.7 ± 10.4 ∆ -0.3 ± 1.5 *r* = -0.100			71.1 ± 15.8 to 77.8 ± 22.2* *r* = 0.192		33.0 ± 4.9 to 35.6 ± 5.6* *r* = 0.177	
							**Main outcome**: **no significant differences between groups**										
Shenoy et al. (2013)	Daily Soy Protein	Randomized controlled study	Total (*n* = 60)	postmenopausal women (45–65 years)	**Duration: 12 weeks**		Muscle strength							bone mineral density		No body composition parameters	
							Isokinetic strength (peak torque)				Maximum power			Radial	Tibial		
							knee flexors (Nm)	knee extensors (Nm)	elbow flexors (Nm)	elbow extensors (Nm)	knee flexors (Nm)						
			ST + P (*n* = 20)	Ø 54.10 ± 6.9 years	**4/ week** resistance training	**Daily**: 40 g of soy protein form: powder to be incorporated into 200–250 ml of milk twice a day	38.9 ± 17.6 to 129.1 ± 47.5*# *r* = 0.811	43.1 ± 17.9 to 106.6 ± 24.2*# *r* = 0.833	19.7 ± 8.9 to 60.1 ± 9.8*# *r* = 0.707	24.5 ± 10.6 to 58.2 ± 7.7*# *r* = 0.872	18.8 ± 8.5 to 64.0 ± 10.8*# *r* = 0.919			3904.9 ± 113.1 to 4369.4 ± 85.3*# *r* = 0.920	3652.5 ± 106.8 to 4333.3 ± 90.3*# *r* = 0.961		
			P (*n* = 20)	Ø 54.55 ± 5.2 years	no supervised training	no supplement	34.6 ± 13.7 to 50.4 ± 17.8* *r* = 0.449	49.1 ± 22.4 to 66.9 ± 26.9* *r* = 0.449	25.1 ± 14.4 to 37.0 ± 16.0* *r* = 0.363	25.7 ± 12.7 to 36.1 ± 13.4* *r* = 0.369	18.6 ± 11.2 to 32.5 ± 11.9* *r* = 0.517			3944.1 ± 57.0 to 4122.6 ± 96.0* *r* = 0.759	3705.5 ± 120.5 to 3985.5 ± 113.1* *r* = 0.701		
			C (*n* = 20)	Ø 54.05 ± 7.1 years			33.9 ± 13.9 to 36.6 ± 13.5* *r* = 0.097	33.9 ± 13.9 to 14.0 ± 47.5* *r* = 0.122	20.0 ± 9.1 to 19.6 ± 8.5 *r* = -0.026	24.8 ± 7.8 to 24.2 ± 7.4 *r* = -0.043	20.2 ± 10.8 to 20.6 ± 11.7 *r* = 0.018			3875.5 ± 108.3 to 3868.5 ± 119.4 *r* = -0.031	3738.1 ± 126.5 to 3747.9 ± 130.4 *r* = 0.038		
							**Main outcome:** **- In comparison to the nutrition (P) and the control (C) group: ** **The training intervention (ST + P) showed a significantly greater increase in all parameters (#)** **- in comparison to the control (C) group:** the non-exercising protein (P) group showed a significant increase in isokinetic strength parameters and BMD parameters (*)										
Weisgarber et al. (2015)	Protein supplement	Randomized double-blind **within subject** study	Total (*n* = 12)	postmenopausal women Ø 57 ± 4.7 years	**Duration: 10 weeks**		body composition							muscle strength			
							SMM				LM			biceps curls (kg)	leg extension (kg)	triceps extension (kg)	leg curl (kg)
							knee flexors (cm)	knee extensors (cm)	elbow flexors (cm)	elbow extensors (cm)	arm (g)		leg (g)				
			ST + P (*n* = 12)		**4/ week** resist. training with training: 2/ week the right side of the body 2/ week the left side	2/week: following each exercise: 40 g of whey **protein** (consumed in 4 × 10 g)	**4.4 ± 0.7 to 4.6 ± 0.6** **∆ +3.0 ± 6.6%** *r* = 0.222	**3.2 ± 1.0 to 3.4 ± 0.8** **∆ +6.4 ± 13.7%** *r* = 0.227	**2.4 ± 0.5 to 2.6 ± 0.6** ∆ +3.2 ± 9.7%* *r* = 0.163	**3.3 ± 0.8 to 3.7 ± 0.6** ∆ +17.2 ± 24.3%* *r* = 0.334	1847.3 ± 419.0 to 1920 ± 419.4 *r* = 0.087		6067.3 ± 975.8 to 6228.9 ± 1082.1 *r* = 0.078	**18.2 ± 3.9 to 1920 ± 419.4** ∆ +34.5 ± 32.6%* *r* = 0.468	59.7 ± 15.3 to 65.2 ± 9.9* ∆ +7.6 ± 11.2%* *r* = 0.321	11.0 ± 1.7 to 20.5 ± 4.2* ∆ +87.7 ± 24.4%* *r* = 0.874	61.8 ± 11.9 to 60.1 ± 7.4 ∆ +0.7 ± 17.0% *r* = -0.021
			ST (*n* = 12)			2/ week: following each exercise: **Placebo**: 30 g of maltodextrin and 10 g of sucrose	4.6 ± 0.7 to 4.6 ± 0.6 ∆ +1.7 ± 10.9% *r* = 0.078	3.3 ± 1.0 to 3.6 ± 0.85 ∆ +2.2 ± 14.8% *r* = 0.074	2.5 ± 0.5 to 2.6 ± 0.4* ∆ +12.0 ± 16.5%* *r* = 0.342	3.3 ± 0.4 to 3.7 ± 0.5* ∆ +15.2 ± 15.2%* *r* = 0.452	1808.3 ± 341.9 to 1853.0 ± 337.9 *r* = 0.065		6162.7 ± 923.5 to 6186.4 ± 1080.3 *r* = 0.012	19.0 ± 4.4 to 22.5 ± 4.1* ∆ +22.6 ± 30.1%* *r* = 0.351	61.0 ± 16.1 to 66.2 ± 13.5* ∆ +9.3 ± 30.1%* *r* = 0.171	11.0 ± 1.7 to 20.2 ± 5.2* ∆ +83.8 ± 28.7%* *r* = 0.825	61.5 ± 12.2 to 62.5 ± 7.1* ∆ +1.9 ± 13.7% *r* = 0.069
							**No significant differences between groups**										

### Amino Acids

In total, three studies [52–54] examined the effect of different amino acids in combination with ST. Two studies examined L-citrulline (LCi), and one focused on branched-chain amino acids (BCAA) supplementation [52–54]. Figueroa et al. [53] reported no significant differences in body composition or muscle strength between ST with and without LCi supplementation. However, the group that received both ST and 6 g of LCi per day showed a significant increase in appendicular lean mass index (from 7.6 ± 0.3 to 7.9 ± 0.3) over time, whereas neither the training-only group (8.1 ± 0.3 to 8.2 ± 0.3) nor the supplementation-only group (7.4 ± 0.3 to 7.5 ± 0.3) demonstrated significant changes. [53] Moreover, Kang et al. [54] reported that the group receiving ST combined with LCi (10 g/day) showed a significantly greater increase in leg curl strength (ST + LCi: 21.8 ± 4.7 kg to 28.7 ± 6.5 kg; ST: 19.8 ± 3.1 kg to 23.8 ± 3.8 kg) and leg lean mass (ST + LCi: 13.4 ± 2.2 kg to 14.0 ± 2.5 kg; ST: 12.6 ± 1.6 kg to 12.4 ± 1.6 kg) compared with the ST-only group. In contrast Bagheri et al. [4, 52, 53]

**Table 4:** Amino acids

Author/Date	Category	Study design	Population		Training	Nutrition	Outcomes													
Bagheri et al (2021)	**BCAA**	Randomized placebo-controlled, double blind study	Total (n = 30)	Postmenopausal women (50–60 years) Ø 56 ± 3.7 years	**Duration: 8 weeks**		Body composition							Muscle strength						
							BW (kg)	BMI (kg/m^2^)	SMM (kg)				BF (%)	Leg press kg)	Chest press (kg)	Quadriceps Strength (kg)		Right Handgrip		Left Handgrip
			ST + BCAA (n = 10)		**3/week** resist. training	On exercise days: 9 g of **BCAA** that contained 4.5 g of leucine, 2.25 g of isoleucine and 2.25 g of valine in capsule form	71.1 ± 5.1 to 72.6 ± 5.1* r = 0.165	28.8 ± 1.9 to 29.5 ± 1.9* r = 0.181	24.3 ± 3.2 to 26.3 ± 3.5* r = 0.285				43.0 ± 4.4 to 39.7 ± 8.2* r = -0.226	**∆ + 5.0°**	**∆ + 4.4°**	36.5 ± 5.4 to 40.4 ± 5.7* r = 0.331		21.6 ± 2.5 to 25.5 ± 2.5*# r = 0.615		20.7 ± 2.5 to 25.6 ± 3.5*# r = 0.617
			ST (n = 10)		No supervised training	On exercise days: isocaloric and isovolumetric **placebo** capsule of identical appearance	71.1 ± 5.1 to 72.6 ± 5.1* r = 0.145	27.8 ± 1.6 to 28.4 ± 1.3* r = 0.200	21.6 ± 1.9 to 23.0 ± 2.2* r = 0.321				42.9 ± 6.3 to 40.9 ± 3.2* r = 0.180	**∆ + 3.4°**	**∆ + 3.6°**	35.0 ± 7.0 to 38.8 ± 5.7* r = 0.283		21.9 ± 2.8 to 24.0 ± 3.2* r = 0.328		21.2 ± 3.5 to 23.6 ± 3.2* r = 0.336
			C (n = 10)				70.1 ± 5.1 to 69.8 ± 5.1 r = -0.029	28.0 ± 1.9 to 28.0 ± 1.9 r = 0.000	21.1 ± 2.5 to 21.1 ± 1.9 r = 0.000				40.7 ± 7.6 to 40.7 ± 9.5 r = 0.00	–-	–-	39.4 ± 5.4 to 37.3 ± 6.3* r = -0.175		20.7 ± 2.8 to 20.5 ± 2.5* r = -0.038		19.8 ± 2.2 to 18.7 ± 3.5 r = -0.177
							**Main outcome: no significant differences between the groups **°no exact data published that is necessary to calculate r													
Figueroa et al (2015)	**L-Citrulline**	Randomized parallel group controlled study	Total (n = 41)	overweight postmenopausal women	**Duration: 8 weeks**		Body composition									Muscle strength				
							BW (kg)	BMI (kg/m^2^)	BF (%)				leg LM (kg)	arm LM (kg)	appendicular LM index	leg strength (kg)		arm strength (kg)		
			WBV + LCi (n = 13)	Ø 58 ± 3 years	**3/ week** whole-body vibration training	6 g/d **LCi** form: capsules	88.3 ± 11.7 to 89.1 ± 12.4 r = 0.033	33.8 ± 3.9 to 34.1 ± 4.3 r = 0.036	47.1 ± 1.3 to 46.2 ± 1.4* r = -0.353				15.1 ± 2.7 to 16.0 ± 2.7 r = 0.164	4.6 ± 0.7 to 4.7 ± 0.9 r = 0.047	7.6 ± 0.3 to 7.9 ± 0.3* r = 0.347	233 ± 51 to 316 ± 95* r = 0.654		55 ± 13 to 56 ± 13 r = 0.023		
			WBV (n = 14)	Ø 58 ± 4 years		**Placebo** containing maltodextrin	89.5 ± 10.6 to 90.0 ± 11.3 r = 0.023	35.0 ± 3.4 to 35.1 ± 4.0 r = 0.013	46.9 ± 1.4 to 46.0 ± 1.3* r = -0.406				15.9 ± 3.0 to 16.0 ± 2.6 r = 0.018	5.0 ± 0.9 to 5.0 ± 1.0 r = 0.000	8.1 ± 0.3 to 8.2 ± 0.3 r = 0.045	238 ± 72 to 316 ± 95* r = 0.414		58 ± 16 to 59 ± 16 r = 0.016		
			LCi (n = 14)	Ø 58 ± 4 years	no supervised training	6 g/d **LCi** form: capsules	83.8 ± 8.4 to 84.4 ± 7.9 r = 0.037	32.7 ± 3.1 to 33.1 ± 2.8 r = 0.067	46.7 ± 1.3 to 46.7 ± 1.3 r = 0.000				14.8 ± 1.6 to 14.9 ± 1.9 r = 0.028	4.6 ± 0.7 to 4.7 ± 0.9 r = 0.061	7.4 ± 0.3 to 7.5 ± 0.3 r = 0.045	227 ± 56 to 238 ± 55 r = 0.099		56 ± 7 to 57 ± 8 r = 0.033		
							**Main outcome: - in comparison to LCi: the WBV group showed a significant greater increase in leg muscle strength (#) the WBV + LCi group showed a significant greater increase in leg LM and leg muscle strength (#) - no significant differences between the training groups**													
Kang et al (2022)	**L-Citrulline**	Randomized, parallel-group, double blind, controlled study	Total (n = 24)	Postmenopausal women (50–75 years)	**Duration: 4 weeks**		Body composition									Muscle strength				
							BW (kg)	BMI (kg/m^2^)	WC (cm)	Visceral FM (kg)			BF (%)	Arm LM (kg)	Leg LM (kg)	Leg press (kg)	LEG extension (kg)		Leg curl (kg)	Calf raise (kg)
			ST + LCi (n = 13)	Ø 62 ± 2 years	**3/ week **slow-velocity low-intensity resistance training	10 g/d **LCi** form: capsules	72.3 ± 10.1 to 72.5 ± 10.5 r = 0.010	29.6 ± 3.9 to 29.5 ± 4.3 r = -0.012	91.5 ± 10.4 to 90.8 ± 8.6 r = -0.036			0.84 ± 0.5 to 0.81 ± 0.5 r = -0.031	42.9 ± 5.0 to 42.0 ± 3.9 r = -0.097	4.3 ± 0.8 to 4.3 ± 0.8 r = 0.013	13.4 ± 2.2 to 14.0 ± 2.5*# r = 0.126	96.4 ± 35.0 to 109.6 ± 32.5* r = 0.192	20.9 ± 5.4 to 27.7 ± 7.5* r = 0.450		21.8 ± 4.7 to 28.7 ± 6.5*# r = 0.511	84.4 ± 29.9 to 102.6 ± 38.9* r = 0.250
			ST (n = 11)	Ø 63 ± 1 year		**Placebo** containing maltodextrin	72.2 ± 12.0 to 72.4 ± 12.4 r = 0.063	29.2 ± 6.1 to 29.4 ± 6.1 r = 0.016	93.6 ± 14.8 to 92.6 ± 14.5 r = -0.032		1.2 ± 0.7 to 1.15 ± 0.8 r = -0.037		29.6 ± 3.9 to 29.5 ± 4.3 r = 0.015	4.1 ± 0.7 to 4.1 ± 0.7 r = 0.007	12.6 ± 1.6 to 12.4 ± 1.6 r = -0.060	88.1 ± 33.2 to 100.2 ± 30.7* r = 0.177	19.6 ± 4.7 to 27.3 ± 7.3* r = 0.498		19.8 ± 3.1 to 23.8 ± 3.8* r = 0.476	78.6 ± 18.7 to 94.4 ± 16.1* r = 0.395
							**Main outcome: ST + LC showed a significantly greater increase in leg LM and leg curl strength than ST only group (#)**													

### Calcium and Vitamin D

Four studies [38, 55–57] investigated the effects of ST in combination with calcium and/or vitamin D supplementation. In three of these studies [38, 55, 57] the participants received 800–1000 mg of calcium/day and two of these also included a daily dose of 400 IU of vitamin D. Haghighi et al. [56] administered 50,000 IU of vitamin D every two weeks without calcium supplementation. All participants in the exercising groups performed ST three times per week. The study periods ranged from 12 weeks to four years.

The four-year longitudinal study by Cussler et al. [55–57] demonstrated that daily supplementation of 1000 mg calcium and 400 IU vitamin D was associated with a significant percentage reduction in BMD in the lumbar region (∆-1.75 %) after one year. In contrast, no decrease was observed in the ST group, which exhibited significant increases in BMD in both the lumbar region (∆-1.15 %) and the femur (∆-2.57 %). In contrast, Kerksick et al. [38] and Haghighi et al. [56] did not show any significant differences between the exercising groups. Only in comparison to the non-exercising groups, Haghighi et al. [56] showed a significantly greater increase in all four muscle strength parameters in the training groups.

Detailed information are provided in Table 5.

**Table 5:** Calcium and Vitamin D

Author/Date	Study design	Population		Training	Nutrition	Outcomes								
Cussler et al (2005)	Randomized controlled study	Total (n = 167)	Postmenopausal women Ø 56.15 ± 4.4 years	**Duration: 4 years **aiming for**: 3/ week **resistance training		Body composition			Bone mineral density				No strength parameters	
						BW (kg)	LBM (kg)	BF (%)	Femur trochanter (g/cm^2^)	Femur neck (g/cm^2^)	Lumbar spine (g/cm^2^)	Total body (g/cm^2^)		
		RT + Ca **high** exercise frequency		Highest exercise frequency (average: 70% of the time)	Aiming for: 800 mg/day of **calcium**	∆ + 0.9 ± 3.1 r = 0.144	**∆ 0.9 ± 1.2#** r = 0.351	**∆ -0.5 ± 2.8#** r = -0.089	**∆ + 0.014 ± 0.033#** r = 0.202	∆ + 0.004 ± 0.037 r = 0.054	**∆ + 0.022 ± 0.046#** r = 0.233	**∆ 0.000 ± 0.018#** r = 0.000		
		RT + Ca **middle** exercise frequency		Middle exercise frequency (average: 30%)		**∆ -0.6 ± 5.3#** r = -0.057	∆ + 0.1 ± 1.6# r = 0.031	**∆ -0.7 ± 4.3#** r = -0.081	**∆ -0.003 ± 0.041#** r = -0.037	∆ -0.005 ± 0.047 r = -0.053	**∆ -0.007 ± 0.055#** r = -0.064	**∆ -0.014 ± 0.026#** r = -0.260		
		RT + Ca **low** exercise frequency		Lowest exercise frequency including 23 controls (average: 5%)		**∆ + 2.4 ± 4–8#** r = 0.243	∆ + 0.4 ± 1.5 r = 0.132	**∆ + 1.7 ± 3.4#** r = 0.243	**∆ -0.007 ± 0.036#** r = -0.097	∆ -0.005 ± 0.042 r = -0.059	**∆ -0.010 ± 0.046#** r = -0.108	**∆ -0.011 ± 0.027#** r = -0.200		
		All groups		All exercise frequencies	** < **800mg/d actual supplemental calcium intake				**∆ -1.8 ± 0.9%**° r = -0.707	**∆ -1.4 ± 0.8%**° r = -0.659	**∆ -2.2 ± 0.6%**° r = -0.808	**∆ -2.5 ± 0.3%**° r = -0.972		
					** > **800mg/d actual supplemental calcium intake				**∆ + 1.3 ± 1.3%**° r = -0.447	**∆ + 1.2 ± 1.4%**° r = -0.394	**∆ + 0.1 ± 1.3%**° r = -0.038	**∆ -1.5 ± 0.5%**° r = -0.832		
						**Main outcome: - in comparison to group middle and low frequency: group high frequency shwoed a significantly greater increase in LBM and BMD (FT, LS and TB) - in comparison to group low frequency: group high frquency and middle showed a significantly decrease in BF and group middle a significantly decrease in BW -in comparison to a high calcium intake: the low group (< 800mg calcium/d) showed a significantly decrease in BMD (FT and TB) °no exact data published that is necessary to calculate r**								
Haghighi et al. (2024)	Randomized, double-blind, placebo-controlled study	Total (n = 44)	Postmenopausal	**Duration: 12 weeks**		Body composition		Muscle strength				No bone mineral density parameter		
						BW (kg)	BMI (kg/m^2^)	leg press (kg)	chest press (kg)	lower body (cm)	upper body (cm)			
		RT + VitD (n = 11)	Ø 55.4 ± 3.8 years	**3/ week **resistance training	Every two weeks: 50.000 IU **vitamin D**	69.7 ± 8.0 to 69.2 ± 7.5 r = -0.032	28.6 ± 3.1 to 28.4 ± 2.9 r = -0.033	31.1 ± 12.8 to 53.1 ± 9.1*# r = 0.694	20.7 ± 8.0 to 34.9 ± 5.7*# r = 0.705	17.3 ± 5.5 to 18.1 ± 4.2*# r = 0.080	256 ± 34 to 298 ± 34*# r = 0.525			
		RT (n = 11)	Ø 55.4 ± 0 years		**Placebo** containing rice flour	74.4 ± 7.8 to 72.5 ± 5.9* r = -0.134	29.8 ± 3.7 to 29.1 ± 2.8* r = -0.104	44.5 ± 10.0 to 63.7 ± 16.0*# r = 0.566	21.6 ± 5.7 to 38.6 ± 5.7*# r = 0.831	15.0 ± 4.5 to 18.5 ± 6.7*# r = 0.284	260 ± 68 to 322 ± 35*# r = 0.466			
		VitD (n = 11)	Ø 57.4 ± 4.8 years	No supervised training	every two weeks: 50.000 IU **vitamin D**	69.0 ± 12.8 to 68.0 ± 11.5 r = -0.039	29.9 ± 5.0 to 29.5 ± 4.5 r = -0.042	27.7 ± 5.9 to 28.4 ± 7.5 r = 0.051	20.7 ± 4.9 to 23.4 ± 8.0 r = 0.190	14.9 ± 2.0 to 14.2 ± 3.3 r = -0.121	226 ± 28 to 230 ± 28 r = 0.071			
		C (n = 11)	Ø 55.8 ± 4.7 years		**Placebo **containing rice flour	75.0 ± 13.9 to 75.1 ± 13.4 r = 0.004	30.1 ± 5.3 to 30.1 ± 5.2 r = 0.003	33.1 ± 3.6 to 34.1 ± 14.7 r = 0.038	27.2 ± 7.0 to 29.0 ± 7.7 r = 0.121	13.2 ± 5.1 to 13.1 ± 5.4 r = -0.010	243 ± 40 to 241 ± 38 r = -0.026			
						**Main outcome: -in comparison to VitD and C: **b**oth RT groups showed a significantly greater increase in all four muscle strength parameters** (#) **no significant differences between the two training groups**								
Kerksick et al (2020)	Randomized, controlled double-blind study	Total (n = 128)	Obese/ overweight postmenopausal Women (50—75 years)	**Duration: 14 weeks**		Body composition				Muscle strength				Bone mineral density
						BW (kg)	FM (kg)	FFM (kg)	BF (%)	Bench press 1RM (kg)	Bench press LV	Leg press 1RM (kg)	Leg press LV	BMC (g)
		RT + Ca (n = 47)		**3/ week **resistance training (30 min.)	800 mg/d **calcium**	92.7 ± 14.6 to 88.4 ± 13.3* r = -0.152	41.2 ± 9.9 to 38.3 ± 9.9* r = -0.145	44.9 ± 5.2 to 44.4 ± 5.5 r = -0.049	47.4 ± 4.6 to 45.7 ± 5.2* r = -0.170	27.6 ± 5.6 to 29.3 ± 5.0* r = 0.158	142 ± 56 to 155 ± 59 r = 0.063	140.3 ± 31.5 to 150.8 ± 33.8* r = 0.158	1535 ± 738 to 1415 ± 581 r = -0.089	1745 ± 239 to 1761 ± 250 r = 0.033
		RT + Ca + D (n = 42)			800 mg/d **calcium**, 400 IU/d **vitamin D**, 300 mg magnesium, 7.5 mg zinc, 2 mg copper and 2 mg manganese	90.4 ± 20.8 to 86.9 ± 20.2* r = 0.085	39.5 ± 12.8 to 35.9 ± 12.5* r = -0.141	44.6 ± 7.9 to 44.8 ± 7.8 r = 0.013	46.2 ± 5.0 to 43.6 ± 5.3* r = -0.244	27.6 ± 7.0 to 31.3 ± 6.5* r = 0.250	169 ± 71 to 161 ± 73 r = -0.055	141.3 ± 45.9 to 157.7 ± 40.1* r = 0.186	1531 ± 844 to 1644 ± 1014 r = 0.060	1755 ± 299 to 1732 ± 298 r = -0.038
		RT (n = 39)			Placebo: 800 mg maltodextrin	88.5 ± 13.4 to 85.7 ± 12.8* r = -0.106	38.2 ± 7.5 to 35.5 ± 7.4* r = -0.178	44.1 ± 6.5 to 44.1 ± 6.1 r = 0.000	46.2 ± 3.4 to 44.3 ± 3.7* r = -0.258	28.2 ± 4.6 to 32.1 ± 6.7* r = 0.312	206 ± 119 to 176 ± 71 r = -0.143	148.8 ± 34.0 to 164.6 ± 40.2* r = 0.206	1720 ± 1284 to 1563 ± 860 r = 0.069	1719 ± 272 to 1698 ± 238 r = -0.041
						**Main outcome no significant differences between the groups**								
Molina et al (2015)	Randomized, controlled study	Total (n = 34)	Postmenopausal	**Duration: 1 year**		Boner mineral density		No strength and bone mineral density parameters						
						Femoral (%)	LUMBAR (%)							
		RT + Ca (n = 7)	Ø 56.6 ± 6.5 years	**3/ week **resistance training	1000 mg/d **Calcium **and 400 IU/d of **Vitamin D**	∆ + 2.57*	∆ + 1.15*							
		Ca (n = 27)	Ø 60.1 ± 10.9 years	no supervised training		∆ -1.68	∆ -1.75*							
						**Main outcome: - In comparison to the Ca group: **the RT + Ca group showed a significant increase in both BMD parameters (*)								

### Creatine Monohydrate

The effects of creatine monohydrate were investigated in four studies [58–61] Two studies went for one year and the other two for 12 weeks, all of them performing ST three times per week. Three studies supplied a daily dose of 0.1 g of creatine/kgBW to the intervention group [58, 59, 61] and one study 5 g of creatine per day [60].

Aguiar et al. showed, after 12 weeks of ST, a greater increase in all muscle strength parameters in the creatine group in comparison to the control group (bench press Cr: Δ+14.3 ± 6.7 %, CO: Δ+9.2 ± 4.4 %; knee extension Cr: Δ+8.6 ± 2.7 %, CO Δ+4.7 ± 1.8 %; biceps curls Cr: Δ+22.3 ± 7.1 %, CO Δ+13.5 ± 3.9 %) [60]. The body composition parameters FFM and SMM likewise only significantly increased in the creatine group but not in control without significant interaction effect. No significant differences were observed for the other body composition parameters. The only comparable outcome parameter of the other 12-week study of Johannsmeyer et al., lat pull down strength, significantly increased in both groups with no differences between the groups. After one year, Candow et al. [58] reported that the supplemented training group showed a significant group difference in lower leg muscle density. The ST group receiving creatine exhibited a significant increase compared with the exercising control group (ST + Cr: 70 [62–67] to 73 [62–67] mg/cm^3^; ST: 69 [62–64, 68, 69] to 70 [62–64, 68, 69] mg/cm^3^). Furthermore, in the study by Chilibeck et al. [59] the exercising group demonstrated a significantly greater decline in femoral neck BMD than the supplemented group. For all other parameters related to body composition, muscle strength and BMD no significant differences were observed across the year-long studies.

Detailed information is provided in Table 6.

**Table 6:** Creatine

Author/Date	Study design	Population					Training			Nutrition	Outcomes																	
Aguiar et al (2013)	Randomized, controlled study	Total (n = 18)		Postmenopausal women			**Duration: 12 weeks**				Body composition									Muscle strength							No BMD parameters	
											Body mass (kg)		FM (kg)		BF (%)		FFM (kg)		SMM (kg)	bench press (kg)		knee extension (kg)				biceps curl (kg)		
		ST + Cr (n = 9)		Ø 64 ± 4 years			**3/ week** resistance training		5 g/d **Creatine** monohydrate form: capsules		60.5 ± 9.4 to 61.2 ± 9.6 r = 0.037		25.4 ± 7.4 to 25.0 ± 7.4 r = -0.027		41.3 ± 5.6 to 40.1 ± 5.7 r = -0,106		35.1 ± 2.3 to 36.2 ± 2.5* r = 0.223		15.5 ± 1.5 to 16.1 ± 1.4* r = 0.202	Δ + 14.3 ± 6.7%*#°		Δ + 8.6 ± 2.7%*#°				Δ + 22.3 ± 7.1%*#°		
		ST (n = 9)		Ø 65 ± 6 years					**Placebo** containing maltodextrin		57.1 ± 7.3 to 57.4 ± 7.5 r = 0,02		22.4 ± 5.8 to 22.6 ± 6.4 r = 0.016		38.8 ± 6.6 to 38.9 ± 7.4 r = 0.007		34.7 ± 4.0 to 34.7 ± 3.8 r = 0.000		15.4 ± 2.6 to 15.5 ± 2.5 r = 0.02	Δ + 9.2 ± 4.4%*°		Δ + 4.7 ± 1.8%*°				Δ + 13.5 ± 3.9%*°		
											**Main outcome: - in comparison to ST: the ST + Cr group showed a significantly greater increase in all muscle strength parameters (#)** and only the ST + Cr group a significant increase in FFM and SMM (*) °no further data published that is necessary to calculate r																	
Candow et al (2021)	Randomized controlled study	Total (n = 22)		Postmenopausal women (> 49 years)		**Duration: 1 year**					Body composition							Bone mineral density									No strength parameters	
											Forearm SMM (mm^2^)	Forearm muscle density (mg/cm^3^)		Lower leg SMM (mm^2^)		Lower leg muscle density (mg/cm^3^)		Distal radius (mg/cm^3^)			Radial shaft (mg/cm^3^)		Distal tibia (mg/cm^3^)		Tibial shaft (mg/cm^3^)			
		ST + Cr (n = 10)				**3/ week** resistance training		0.1g/kg/d **Creatine** monohydrate form: powder			2661 (2274–3048) to 2782 (2426–3138)* r = 0.115	74 (73–75) to 74 (73–75) r = 0.000		6198 5501–6896) to 6023 (5313–6732)* r = -0.089		**70 (68–73) to 71 (68–73)#** r = 0.142		285 (253–317) to 279 (248–311) r = -0.138			1110 (1088–1132) to 1114 (1091–1137) r = 0.027		274 (253–295) to 272 (252–293)* r = -0.025		1081 (1059–1102) to 1082 (1061–1103) r = -0.024			
		ST (n = 12)						**Placebo** containing maltodextrin			2956 (2603–3309) to 3000 (2675–3324)* r = 0.041	74 (73–75) to 74 (73–75) r = 0.000		7071 6406–7736) to 6680 (6003–7356)* r = -0.182		69 (66–71) to 68 (66–70) r = -0.137		283 (254–312) to 277 (248–305) r = -0.031			1108 (1088–1129) to 1103 (1082–1124) r = -0.025		284 (264–304) to 281 (262–301)* r = 0.016		1068 (1048–1089) to 1074 (1054–1094) r = 0.044			
											**Main outcome: the ST + Cr group showed a significant increase in lower leg muscle density compared to ST only (#)**																	
Chilibeck et al (2015)	Randomized, controlled study	Total (n = 33)		Postmenopausal women		**Duration: 1 year**					Body composition							Muscle strength					Bone mineral density					
											LBM (kg)	Triceps SMM (thickness) (cm)		Quadriceps SMM (thickness) (cm)		Gastrocnemius SMM (thickness) (cm)		Bench press (kg)			Squat press (kg)		Femoral neck (mg/cm^3^)		Total hip (mg/cm^3^)		Lumbar spine (mg/cm^3^)	Total body (mg/cm^3^)
		ST + Cr (n = 15)		Ø 57 ± 4 years		**3/ week** resistance training		0.1g/kg/d **Creatine** monohydrate form: powder			∆ -1.0 (-2.1 to 0.8)* r = -0.224	∆ + 0.3 (-0.1 to 0.7)* r = 0.186		∆ + 0.2 (-0.3 to 0.6) r = 0.101		∆ + 0.5 (-0.1 to 1.2)* r = 0.206		∆ + 18 (10 to 25)* r = 0.495			∆ + 54 (29 to 79)* r = 0.480		∆ -0.010 (-0.025 to 0.005) r = -0.166		∆ -0.015 (-0.037 to 0.007)* r = -0.170		∆ + 0.005 (-0.017 to 0.028) r = 0.057	∆ -0.008 (-0.018 to 0.002) r = -0.198
		ST (n = 18)		Ø 57 ± 7 years				**Placebo** containing maltodextrin			∆ -1.3 (-2.3 to -0.3)* r = -0.288	∆ + 0.4 (0 to 0.8)* r = 0.225		∆ + 0.3 (-0.1 to 0.8) r = 0.171		∆ + 0.5 (-0.1 to 1.1)* r = 0.189		∆ + 11 (5 to 18)* r = 0.390			∆ + 50 (29 to 71)* r = 0.482		**∆ -0.030 (-0.044 to 0.017)#** r = -0.444		∆ -0.016 (-0.036 to 0.004)* r = -0.182		∆ -0.007 (-0.027 to 0.014) r = -0.081	∆ + 0.002 (-0.007 to 0.012) r = 0.051
											**Main outcome: the ST group showed a significantly greater decrease in femoral neck BMD than ST + Cr group**																	
Johanns meyer et al. (2016)	Randomized, controlled study	Total (n = 14)	Postmeno-pausal women		**Duration: 12 weeks**						Muscle strength													Body composition and other muscle strength parameters were not differentiated between males and females				
											Lat. pull down (kg)																	
		ST + Cr (n = 7)	Ø 58 ± 3 years		**3/ week **resistance training				0.1 g/kg/d **Creatine** monohydrate form: powder		**39.3 ± 5.9 to 45.8 ± 7.2** r = 0.439*																	
		ST (n = 7)	Ø 57.6 ± 5 years						**Placebo** containing maltodextrin		**38.2 ± 9.7 to 45.6 ± 10.6** r = 0.342*																	
											**No significant differences between groups**																	

### Zataria Multiflora

The effects of *Zataria multiflora* (Z) supplementation in combination with strength training were examined in two studies involving postmenopausal women over an eight-week period [70, 71]. Ghanbari-Niaki et al. observed a significant group difference in SMM and FM between ST + Z and control group, but not when compared ST only or Z only. The ST + Z group showed a reduction in FM from 21.0 ± 1.4 kg to 18.0 ± 1.1 kg compared with 20.1 ± 2.1 kg to 17.0 ± 1.9 kg in the ST group and from 19.9 ± 1.9 kg to 18.6 ± 1.4 kg in the Z group. In addition, both training groups showed a significant increase in SMM over time whereas no significant change was observed in the Z only group (ST + Z: 28.0 ± 1.9 kg to 29.6 ± 1.9 kg; ST: 26.8 ± 2.8 kg to 28.4 ± 2.2 kg; Z: 26.5 ± 2.5 kg to 26.8 ± 2.1 kg) [72]. Tayebi et al. reported similar results for BF and SMM. A significant decrease in BF over time was observed for ST + Z at both training intensities (35 % and 55 %) as well as in the Z only group without training. Moreover, significant group differences were found between each training group and the control group, whereas no differences were detected between the training groups. In addition, only the training groups exercising at an intensity of 55 % showed a significant increase in SMM over time [71].

Detailed information is provided in Table 6.

### Omega-3 Fatty Acids (“Fish Oil”)

Only one study has investigated the combined effects of ST and omega-3 fatty acid (FO) supplementation. Felix-Soriano et al. [73] evaluated the effects of FO supplementation combined with ST performed twice per week. Participants received a daily dose of 1650 mg docosahexaenoic acid (DHA) and 150 mg eicosapentaenoic acid (EPA) alongside a Mediterranean diet. Over the 16-week intervention, no significant differences were observed between the two training groups; significant differences were only noted in comparison with the non-exercising groups (see Sect. 3.10). Both training groups showed significant increases in muscle strength in the upper body (ST + FO: ∆+0.11 ± 0.05 kg/kg LBM; ST: ∆+0.08 ± 0.06 kg/kg LBM, FO: ∆+0.02 ± 0.03 kg/kg LBM; C: ∆+0.02 ± 0.03 kg/kg LBM), lower body (ST + FO: ∆+1.0 ± 0.8 kg/kg LBM; ST: ∆+0.8 ± 0.6 kg/kg LBM, FO: ∆+0.3 ± 0.4 kg/kg LBM; C: ∆+0.0 ± 0.3 kg/kg LBM), and in lower limb muscle quality (ST + FO: ∆+2.0 ± 1.3 kg/kg LBM; ST: ∆+1.3 ± 1.2 kg/kg LBM, FO: ∆+0.4 ± 0.8 kg/kg LBM; C: ∆-0.4 ± 0.8 kg/kg LBM), compared with the control group. A significant decrease in BMC over time was observed in the FO and control groups, but not in either training groups (ST + FO: ∆-1.4 ± 32.8 g; ST: ∆ -1.4 ± 30.3 g, FO: ∆-17.5 ± 20.2 g; C: ∆-27.6 ± 17.4 g).

Detailed information is provided in Table 7.

### Shatavari

The potential performance-enhancing effect of Shatavari extract was conducted in one study by Greed et al. [68] Participants consumed a daily dose of 1000 mg of *Shatavari* extract over eight weeks, while performing ST twice per week. No significant differences were observed between the Shatavari and the placebo groups for all strength-related parameters. Body composition and BMD were not assessed.

Detailed information is provided in Table 7.

**Table 7:** Others

Author/Date	Category	Study design	Population			Training		Nutrition		Outcomes												
Felix-Soriano et al (2021)	**Fish oil**	Randomized, parallel-group, double blind, controlled study	Total (n = 71)	Overweight/obese postmenopausal women 55–70 years		**Duration: 16 weeks**				Body composition									Muscle strength			Bone mineral density
										BW (kg)	WC (cm)		Waist to hip ratio	BF (%)		Visceral FM (kg)	LBM (%)		CHEST press 1RM (kg/kg LBM)	Leg press 1RM (kg/kg LBM)	Lower limbs muscle quality	BMC (g)
			ST + FO (n = 16)	Ø 58.13 ± 3.14 years		**2/ week** resistance training		**Omega-3 fish oil**: 1650 mg/d DHA + 150 mg/d EPA form: capsules ** + Mediterranean diet**		Pre 80.6 ± 6.6 ∆ -2.7 ± 3.5* r = -0.361	Pre 93.9 ± 7.7 ∆ -4.0 ± 3.7* r = -0.476		Pre 0.83 ± 0.06 ∆ -0.02 ± 0.03 r = -0.316	Pre 46.7 ± 2.9 ∆ -2.1 ± 2.4* r = -0.404		Pre 1.2 ± 0.5 ∆ -0.1 ± 0.2* r = -0.252	Pre 50.5 ± 2.7 ∆ -2.0 ± 2.3* r = 0.395		**Pre 0.44 ± 0.05** ∆ + 0.11 ± 0.05*# r = 0.729	**Pre 2.0 ± 0.3** ∆ + 1.0 ± 0.8*# r = 0.563	**Pre 10.9 ± 1.3** ∆ + 2.0 ± 1.3*# r = 0.595	Pre 2240.3 ± 258.4 ∆ -1.4 ± 32.8 r = -0.021
			ST (n = 20)	Ø 58.95 ± 3.46 years				**Placebo** containing olive oil form: capsules ** + Mediterranean diet**		Pre 77.8 ± 7.9 ∆ -2.2 ± 2.4* r = -0.420	Pre 92.7 ± 5.5 ∆ -3.01 ± 1.8* r = -0.641		Pre 0.84 ± 0.06 ∆ -0.00 ± 0.04 r = 0.0	Pre 47.1 ± 4.0 ∆ -1.8 ± 1.5* r = -0.508		Pre 1.3 ± 0.5 ∆ -0.1 ± 0.2* r = -0.292	Pre 50.2 ± 3.8 ∆ -1.7 ± 1.4* r = 0.511		**Pre 0.45 ± 0.08** ∆ + 0.08 ± 0.09*# r = 0.430*	**Pre 2.2 ± 0.4** ∆ + 0.8 ± 0.6*# r = 0.561	**Pre 12.0 ± 1.8** ∆ + 1.3 ± 1.2*# r = 0.476*	Pre 2156.1 ± 231.2 ∆ -1.4 ± 30.3 r = 0.023
			FO (n = 15)	Ø 58.0 ± 2.78 years		no supervised training		**Omega-3 fish oil** ** + Mediterranean diet**		Pre 80.3 ± 8.5 ∆ -2.7 ± 2.5* r = -0.473	Pre 95.0 ± 7.6 ∆ -3.2 ± 2.9* r = -0.472		Pre 0.85 ± 0.06 ∆ -0.01 ± 0.01* r = -0.447	Pre 45.6 ± 2.4 ∆ -1.6 ± 1.3* r = -0.508		Pre 1.4 ± 0.4 ∆ -0.1 ± 0.1* r = -0.366	Pre 51.5 ± 2.2 ∆ -1.5 ± 1.3* r = 0.509		**Pre 0.44 ± 0.10** ∆ + 0.02 ± 0.03* r = 0.358	**Pre 2.3 ± 0.5** ∆ + 0.3 ± 0.4* r = 0.408	**Pre 11.9 ± 2.2** ∆ + 0.4 ± 0.8* r = 0.238	Pre 2366.3 ± 332.8 ∆ -17.5 ± 20.2* r = -0.398
			C (n = 20)	Ø 58.75 ± 3.39 years				**Placebo** ** + Mediterranean diet**		Pre 76.7 ± 5.0 ∆ -2.7 ± 2.9* r = -0.411	Pre 93.1 ± 4.5 ∆ -3.5 ± 2.6* r = -0.550		Pre 0.84 ± 0.04 ∆ -0.01 ± 0.01* r = -0.447	Pre 47.4 ± 3.4 ∆ -2.3 ± 1.1* r = -0.706		Pre 1.3 ± 0.4 ∆ -0.2 ± 0.2* r = -0.466	Pre 49.8 ± 3.2 ∆ -2.0 ± 1.0* r = 0.715		Pre 0.44 ± 0.08 ∆ + 0.02 ± 0.03 r = 0.289	Pre 2.2 ± 0.4 ∆ 0.0 ± 0.3 r = 0.051	Pre 11.6 ± 3.4 ∆ -0.4 ± 0.8 r = -0.252	Pre 2152.7 ± 308.1 ∆ -27.6 ± 17.4* r = -0.622
										**Main outcome: - in comparison to the FO and C group: **the training groups significantly prevented BMD reduction and the training groups showed a significantly greater increase in muscle strength and muscle quality **- no significant differences between the training groups**												
Ghanbari-Niaki et al (2018)	**Zataria multiflora**	Randomized, parallel-group, double blind, controlled study	Total (n = 48)	Postmenopausal women		**Duration: 8 weeks**				Body composition						No strength and bone mineral density parameters						
										BW (kg)	BMI (kg/m^2^)		FM (%)	SMM (kg)								
			ST + Z (n = 12)	Ø 53.8 ± 6.0 years		**3/ week** resistance training		500 mg/d **Z. multiflora**		69.9 ± 5.7 to 67.0 ± 4.0* r = -0.275	27.6 ± 2.7 to 26.4 ± 2.1* r = -0.237		21.0 ± 1.4 to 18.0 ± 1.1*# r = -0.762	28.0 ± 1.9 to 29.0 ± 1.9* r = 0.278								
			ST (n = 12)	Ø 58.03 ± 4.7 years				**Placebo** containing wheat flour		67.1 ± 7.2 to 64.3 ± 5.6* r = -0.209	26.6 ± 3.1 to 25.5 ± 2.3*# r = -0.194		20.1 ± 2.1 to 17.0 ± 1.9*# r = -0.599	26.8 ± 2.8 to 28.4 ± 2.2* r = 0.299								
			Z (n = 12)	Ø 54.4 ± 3.9 years		No supervised training		500 mg/d **Z. multiflora**		66.4 ± 10.9 to 65.9 ± 5.1* r = -0.026	**25.6 ± 2.2 to 25.4 ± 1.6#** r = -0.051		19.9 ± 1.9 to 18.6 ± 1.4*# r = -0.356	26.5 ± 2.5 to 26.8 ± 2.1 r = 0.064								
			C (n = 12)	Ø 56.5 ± 4.2 years				**Placebo** containing wheat flour		68.7 ± 13.3 to 68.7 ± 4.7 r = 000	27.9 ± 2.2 to 27.9 ± 1.8 r = 000		20.6 ± 1.7 to 20.5 ± 1.6 r = -0.030	27.6 ± 2.5 to 27.6 ± 2.3 r = 000								
										**Main outcome: - in comparison with C: The Z and ST group has a significant difference in BMI and FM compared to C group (#) ST + Z group has a significant difference in FM compared to C group (#) -in comparison to Z: ST + Z group showed a significantly greater decrease in FM and a significantly greater increase in SMM (#) -no significant differences between the training groups**												
Tayebi et al (2019)	**Zataria multiflora**	Randomized, parallel-group, double blind, controlled study	Total (n = 72)	Postmenopausal women		**Duration: 8 weeks**				Body composition						No strength and bone mineral density parameters						
										BW (kg)	BMI (kg/m^2^)		BF (%)	SMM (kg)								
			ST 55 + Z (n = 12)	Ø 53.8 ± 6 years		**3/ week** resistance training intensity: **55%** of 1RM		500 mg/d **Z. multiflora**		69.9 ± 5.7. to 67.0 ± 4.0* r = -0.275	27.6 ± 2.7 to 26.4 ± 2.1* r = -0.237		21.0 ± 1.4 to 18.0 ± 1.1*# r = -0.762	**28.0 ± 1.9 to 29.5 ± 1.9 + #** r = 0.367								
			ST 55 (n = 12)	Ø 58.0 ± 4.7 years				**Placebo**		67.1 ± 7.2 to 64.3 ± 4.0* **r = -0.209**	27.6 ± 2.7 to 25.6 ± 2.3* r = -0.194		20.1 ± 1.4 to 18.0 ± 1.1*# r = -0.599	26.8 ± 2.8 to 28.4 ± 2.2* r = 0.299								
			ST 35 + Z (n = 12)	Ø 55.8 ± 4.5 years		**3/ week** resistance training intensity: **35%** of 1RM		500 mg/d **Z. multiflora**		66.8 ± 6.5 to 64.4 ± 5.0* r = -0.199	27.6 ± 2.7 to 26.6 ± 1.9* r = -0.204		20.0 ± 1.9 to 17.5 ± 1.4*# r = -0.591	26.7 ± 2.6 to 27.8 ± 2.0 r = 0.227								
			ST 35 (n = 12)	Ø 57.7 ± 3.6 years				**Placebo**		69.3 ± 6.7 to 67.5 ± 3.9 r = -0.153	27.8 ± 2.9 to 27.0 ± 1.7 r = -0.157		20.7 ± 2.0 to 18.7 ± 1.2*# r = -0.498	27.7 ± 2.7 to 28.7 ± 1.6 r = 0.208								
			Z (n = 12)	Ø 54.4 ± 3.9 years		no supervised training		500 mg/d **Z. multiflora**		66.4 ± 6.4 to 65.9 ± 5.1 r = -0.043	25.6 ± 2.2 to 25.4 ± 1.6 r = -0.051		19.9 ± 1.9 to 18.6 ± 1.4* r = -0.356	26.5 ± 2.5 to 26.8 ± 2.1 r = 0.064								
			C (n = 12)	Ø 56.5 ± 4.2 years				**Placebo**		68.7 ± 5.9 to 68.7 ± 4.7 r = 0.000	27.9 ± 2.2 to 27.9 ± 1.8 r = 0.000		20.6 ± 1.7 to 20.5 ± 1.6 r = -0.030	27.6 ± 2.5 to 27.6 ± 2.3 r = 0.0								
										**Main outcome no significant differences were observed between the groups with the same training intensity**												
Greed et al. (2025)	**Shatavari**	Randomized, parallel-group, double blind, controlled study	Total (n = 22)		Postmenopausal women		**Duration: 8 weeks**			Muscle strength								No body composition and bone mineral density parameter				
										Knee extension (kg)		Leg press (kg)			Hand grip strength (kg)							
			SH (n = 12)		Ø 62.7 ± 5.3 years		2/ week resistance training		1000mg/d Shatavari	59.8 ± 3.5 to 73.8 ± 12.3* r = 0.538		138.7 ± 14.0 to 206.3 ± 42.1* **r = 0.673**			22.9 ± 3.8 to 24.1 ± 4.6 **r = 0.140**							
			PL (n = 11)						Placebo	59.8 ± 9.2 to 70.3 ± 12.5* **r = 0.424**		121.0 ± 29.5 to 180.2 ± 47.5* **r = 0.580**			25.9 ± 4.4 to 26.9 ± 4.8 **r = 0108**							
										**Main outcome no significant differences were observed between the groups**												

AHA = American Heart Association; BCAA = branched-chain amino acids; BF% = Body Fat Percentage; BMD = Bone Mineral Density; BMI = Body Mass Index; BRMR = Baseline Resting Metabolic Rate; BW = Body Weight; C = Control; Ca = Calcium; CR = Caloric Restriction; Cr = Creatine; FFM = Fat Free Mass; FM = Fatt Mass; FO = Fish Oil; HC = Hip Circumference; LBM = Lean Body Mass; LCi = L-Citrulline; SMM = Skeletal Muscle Mass; SH = shatavari; NIS = Nutritional Information Session; P = Protein; PAF = Physical Activity Factor; ST = Strength Training; VitD = Vitamin D; WBV = Whole-body vibration; WC = Waist Circumference; Z = Zataria multiflora; 1RM = One-Repetition-Maximum; r = Pearson correlation coefficient r; * = significant time change; # = significant differences in comparison to other group

### Strength Training vs. Non-exercising Control

When comparing exercising and non-exercising groups, several significant differences were observed, except in control groups following caloric restriction. Bagheri et al. [52] reported significant increases in BW and BMI in the training groups compared with the non-exercising controls, whereas Ghanbari-Niaki et al. [70] observed significant decreases relative to the unchanged control group. All other included studies did not report comparable effects. Regarding body composition parameters, all studies including the two mentioned above showed similar effects with a significant reduction in BF [52, 55, 71], FM [51, 70] and WC [46] and a significant increase in SMM [46, 51, 52, 70, 71] or LBM [55] compared with non-exercising control groups. In terms of muscle strength, all ST groups demonstrated significant improvements compared to the control groups. Eight studies [32, 42, 49, 51–53, 56, 73] assessed a total of seven different exercises for the lower body while five studies [49, 51, 52, 56, 73] evaluated five different exercises for the upper body.

Finally, four studies [49, 55, 57, 73] showed significant differences in BMD. All ST groups showed a greater increase compared to the non-exercising control groups.

More detailed information is provided in Tables 2, 3, 4, 5, 6 and 7.

## Discussion

This systematic review aimed to examine the additive effects of nutritional strategies in combination with ST on body composition, strength capacity, and BMD in postmenopausal women with an average age below 65 years. In total, 34 studies comprising 1,541 participants were identified and analyzed. The interventions included CRD (*n* = 11), high-protein diets (*n* = 3), protein supplementation (*n* = 6), and the use of amino acids (*n* = 3), calcium (*n* = 3), vitamin D (*n* = 3), creatine (*n* = 4), omega-3 fatty acids (*n* = 1), zataria multiflora (*n* = 2) and shatavari (*n* = 1).

### Benefits of Strength Training and Dose Responses

ST generally exerts a significant positive influence on body composition, strength capacity, and BMD. Systematic ST has been shown to reduce FM or BF and increase SMM or LBM. Regarding training frequency, initial evidence from one study suggests that performing two sessions per week with free weights at low (50–60% of 1-RM) to moderate intensities (70–80% of 1-RM) over a 10-week period may be insufficient to elicit significant changes in body composition [45]. In comparison, the same training protocol demonstrated an increase in FFM and SMM in premenopausal women [45]. In contrast, Félix-Soriano et al. [73] reported a significant increase in LBM following a 16-week training intervention with two sessions per week. These findings, however, should be interpreted with caution, as a similar increase was also observed in the control group that did not participate in ST. [73] However, regarding strength capacity, one study found that performing ST twice per week led to significant improvements in squat and bench press strength in postmenopausal women, comparable to those observed in premenopausal women [45]. 

Studies implementing three or four training sessions per week showed significant improvements in body composition, muscle strength and BMD [32, 33, 36, 37, 39, 41, 43, 44, 46–52, 55–57, 70, 71]. Concerning training intensity, individual studies suggest that higher intensities may elicit a greater effect than lower intensities [55, 71]. However, definitive conclusions on dose-response relationships cannot be drawn due to the limited number of studies and lack of formal statistical analyses. Current reviews on the effects of ST alone also report inconsistent findings. A meta-analysis by González-Gálvez et al. [18] indicated that three 60 min training sessions per week, significantly improved lower and upper body strength in postmenopausal women. However, these findings were not corroborated by Thomas et al. [8] and Borde et al. [69] Notably, Borde et al. [69] observed significant changes in muscle morphology with only two training sessions per week. Comparisons between younger and older adults further suggests that younger individuals derive greater benefits from higher training frequencies than older individuals.

Regarding the effects of ST on BMD, a meta-analysis by Shojaa et al. [16] reported that even one training session per week is sufficient to increase BMD. However, the authors noted several limitations, including that some studies failed to observe significant differences in BMD across varying training frequencies. In contrast, other studies have consistently demonstrated significantly greater improvements in lumbar and hip BMD when training frequencies of two or more sessions per week are employed [16]. 

### Caloric Restriction

Sarcopenic obesity is considered a potential risks associated with menopause [14], and dietary interventions may represent a logical strategy to enhance the beneficial effects of ST on body composition. A meta-analysis by Cheng et al. [62] further demonstrated that dietary interventions were more effective than exercise alone in reducing BW and body composition parameters in peri- and postmenopausal women. In this review similar effects were observed when combining CRD and ST. An additional daily calorie deficit between 250 and 750 kcal significantly contributes to reduction in body composition, such as BMI, FM or BF parameters [32, 35, 36, 38, 42]. Furthermore, compared CRD alone LBM, including SMM, can be maintained through ST [32–36]. ST also increases daily energy expenditure, meaning that participants in CRD + ST interventions experience a greater actual energy deficit than the prescribed 250 kcal. The continuous activation of SMM can further enhance long-term energy expenditure, thereby promoting FM reduction while preserving SMM [63]. 

Regarding muscle strength, combining ST with CRD may reduce strength gains when the caloric deficit is excessive, as demonstrated in two studies targeting a weight loss of 0.5–1 kg/week [32, 42]. Furthermore, the additional benefits of ST on body composition parameters appear diminished under excessive caloric restriction, particularly when the deficit is not individualized according to initial BW [32–36, 42].

In terms of BMD, the study of Kerskick et al. [38] (PEDro: 9) showed no significant effects of a CRD (1600 kcal/day) on BMD. In contrast a previous investigation by Svendsen et al. [64] observed a significant decrease in BMD in postmenopausal women after CRD combined with concurrent training. Examining the intervention groups’ calorie intake, the groups reduced their daily energy intake by more than 800 kcal/day (daily intake diet: _~_1093 kcal/day; diet + exercise: _~_1051 kcal/day), which may be insufficient to preserve BMD. These findings suggest that an excessively restrictive CRD can attenuate the beneficial effects of training and lead to a significant reduction in BMD. Likewise, Seimon et al. [65] reported a greater decrease in total hip BMD in postmenopausal women subjected to severe energy restriction (65–75% of estimated energy expenditure) compared with those under moderate restriction (25–35% of estimated energy expenditure). Collectively, these findings indicate that excessively restrictive CRD may reduce BMD, even when physical activity is systematically implemented.

### Protein

Proteins are macronutrients that are metabolized into amino acids within the body [66]. Amino acid availability serves as a key regulatory factor in muscle protein metabolism with improvements in muscular strength and body composition resulting from ST, depending on a predominance of protein anabolism over catabolism [67]. Accordingly, protein intake following exercise can maximize protein synthesis and offset protein catabolism [74]. Consistent with this, studies with healthy adults demonstrated that protein supplementation enhances muscle strength and hypertrophy when combined with ST [25].

When evaluating the effects of protein supplementation, distinctions must be made regarding the form and timing of intake. In this review, three studies provided protein supplementation only immediately after each exercise session [44, 45, 50]. Notably, one of these studies [45] implemented ST only twice per week, which may explain the absence of significant effects, as discussed previously. Additionally, Weisgarber et al. [50] employed a within-participants design alternating between placebo and whey protein supplementation per day of exercise while participants performed unilateral ST. No significant differences were observed between sides of the body of the same participants. The only comparable study by Holm et al. [44] likewise did not reveal significant differences between the two exercising groups overall, but did report a significant time-related increase in LBM and muscle strength in the supplemented training group. When total protein intake was examined, the supplemented group consumed an average of 1.1 g/kgBW/day throughout the intervention, compared with 0.9 g/kgBW/day in the non-supplemented group. Similarly, regarding protein timing, no significant differences were observed between consuming the same amount of protein immediately after exercise or six hours later [43]. This finding is consistent by a meta-analysis of Wirth et al. [75], which reported no additional benefits of a specific protein timing on LBM and muscle strength.

Regarding plant-based protein source, three studies provided daily soy protein supplementation [46, 47, 49]. Of these Shenoy et al. [49] (PEDro: 7) compared the supplemented training group only with non-exercising groups, precluding conclusions about the additional effects of protein supplementation in combination with ST. Maesta et al. [46] (PEDro: 7) did not observe significant differences between the training groups overall, although the supplemented training group demonstrated higher effect sizes for reductions in WC and increases in SMM. Notably, total daily protein intake was not reported in any group. In contrast, Orsatti et al. [47] (PEDro: 9) reported a significantly greater increase in three of four strength parameters in the supplemented training group which consumed an average of 1.4 g/kgBW/day compared with 1.0 g/kgBW/day in the non-supplemented training group.

Additionally, Ioannidou et al. [51] (PEDro: 8) demonstrated that a high-protein diet al.one, providing at least 2.5 g/kg FFM (1.8 g/kgBW/day), significantly improved strength parameters. When combined with ST, the high-protein group (1.7 g/kgBW/day) exhibited greater absolute gains in SMM (ST + P: ∆+1.4 ± 0.9 kg; ST alone: ∆+1.2 ± 1.3 kg), although these differences did not reach statistically significance and should be interpreted with caution. The authors also reported a significant ALT enzyme activity with the high-protein diet. While, there is currently no evidence of adverse effects of a high-protein diet in younger populations, further research in older women is needed to draw definitive conclusions [76, 77]. Current meta-analyses by Kuo et al. [78] support an additive effect of a high-protein diet and are consistent with the current recommendations of the International Society of Sports Nutrition [77]. However, among postmenopausal women, only two high-quality studies (PEDro: 8 and 9) have investigated a high-protein diet (>1.4 g/kgBW/day) in combination with ST, and the evidence from previous studies does not yet clearly confirm benefits in this population [47, 51].

###  Amino Acids

Amino acids can be classified as proteinogenic or non-proteinogenic. Proteinogenic amino acids acts as substrates for protein biosynthesis in animal cells, whereas non-proteinogenic amino acids do not participate in this process [79]. In postmenopausal women, supplementation with proteinogenic BCAAs does not appear to provide additional benefits for body composition or muscle strength when total daily protein intake is comparable between groups [52]. Although enhanced post-exercise muscle recovery has been reported in high-intensity endurance athletes and trained males [72, 80], such effects have not been observed in this population.

While LCi, a non-proteinogenic amino acid, may mechanistically increase nitric oxide bioavailability and improve muscle oxygenation [72, 81–84],only two small high-quality RCTs (PEDro: 8 and 9) in postmenopausal women have been conducted. The study of Kang et al. suggests an additional increase in LM und strength parameters in supplemented, strength-trained women. Figuerora et al. showed no significant differences between the supplemented and non-supplemented strength-trained women, however the performed ST was WBV. The results may therefore be limited by the effectiveness of this training method. [53, 54] Current evidence in postmenopausal women is apparently insufficient to provide clear recommendations and should be considered preliminary. Further studies are warranted to allow more definitive conclusions.

### Calcium and Vitamin D

Vitamin D and calcium are essential for maintaining bone health, and deficiencies in either represent significant risk factor for osteoporosis development [85]. Vitamin D is primarily synthesized in human skin upon exposure to sunlight, but modern lifestyle patterns often limit sun exposure, making dietary sources (e.g., vitamin D2) increasingly important [86]. Once metabolized and activated, vitamin D (1,25-dihydroxyvitamin D) enhances calcium absorption in the intestine, stimulates calcium uptake by osteoblasts, and promotes osteoclast maturation [87, 88]. Calcium constitutes a major structural component of bone (~ 99%) and also plays essential roles in cellular signaling, nerve impulse transmission, and muscle contraction. Inadequate daily calcium intake forces the mobilization of calcium from bone, and prolonged deficiency can lead to decreased BMD [88–90]. 

Regarding the influence of calcium and vitamin D in combination with ST in postmenopausal women, four studies (PEDro: 2, 6, 9, 10) were identified in this review. The findings indicate that a calcium application exceeding 800 mg/day has no additive effects on body composition, strength capacity and BMD [38, 55–57]. However, the study by Cussler et al. [21–23, 55, 56]. However, a precise minimum effective dosage below 800 mg/day cannot currently be established for postmenopausal women. Nonetheless, existing evidence suggests that ST remains the primary factor in preserving BMD in this population.

Concerning vitamin D supplementation combined with ST, no significant effects were observed on body composition, strength capacity, or BMD [38, 56, 57] These results are consistent with previous research indicating that chronic vitamin D supplementation does not significantly influence BMD [24] and are further supported by studies examining combined calcium and vitamin D supplementation in premenopausal women [38]. Based on the limited data on calcium and vitamin D supplementation combined with ST, it appears that supplementation of both substances has no additive effect on body composition, strength and BMD. Importantly, however, a daily calcium intake of at least_~_800 mg/day appears necessary, as lower intakes may contribute to reductions in BMD [55].

### Creatine Monohydrate

Creatine supplementation may mechanistically reduce muscle fatigue and improve performance by prolonging the energy contribution of the phosphocreatine system [91–93] and has been shown to be beneficial in enhancing strength, LBM and BMD in other populations, such as younger and older adults [58, 94–98]. Evidence in especially postmenopausal women however, is limited to four small, high-quality RCTs (PEDro: 9–10) [58–61]. No clear effects on body composition, strength, or BMD were observed [58, 59, 61]. Potential explanations for the lack of observed effects, besides the limited data available, may include that also in other populations only small to moderate effects could be achieved through creatine supplementation [94, 98–103]. Furthermore, two included studies in this review lasted at least one year and therefore it is plausible that ST was not structured into systematic macrocycles. Consequently, participants may have engaged in continuous, unsystematic training, thereby limiting potential adaptive responses. Recent reviews investigating creatine supplementation in women were also unable to draw definitive conclusions regarding postmenopausal populations [104, 105]. These considerations underscore the need for further research on structured, intensive ST combined with creatine supplementation to clarify its potential benefits in postmenopausal women.

### Zararia Multiflora

For supplementing Zataria multiflora only two studies [70, 71] (PEDro: 8 and 9) were identified. Zataria multiflora is a plant used in traditional Iranian medicine, which is rich in antioxidative compounds, including thymol and carvacrol [106]. Previous research indicates that Zataria multiflora supplementation exhibits concentration-dependent radical-scavenging activity against 1,1-diphenyl-2-picryl-hydrazyl free radicals [107]. However, when examining the potential additive effects of Zataria multiflora supplementation in postmenopausal women undergoing ST, no significant differences were observed between the supplemented and non-supplemented exercise groups [70, 71]. 

### Omega-3 Fatty Acids (“Fish Oil”)

Only one study [73] (PEDro: 8) investigated the additional effects of FO supplementation in postmenopausal women performing ST, which limits the generalizability of its findings. The omega-3 fatty acids EPA and DHA have been shown to reduce mitochondrial oxidant emissions, enhance muscle protein synthesis, and improve anabolic responses to exercise in older adults aged 65 and above [108]. In older women, omega-3 supplementation has been associated with increases in SMM and muscle strength in several studies [109–112]. However, in the present no significant differences were observed between the supplemented and non-supplemented exercises group. Further studies specifically targeting postmenopausal women are needed to enable more definitive recommendations and to confirm the findings of previous research in this population.

### Shatavari

Shatavari is an Ayurvedic herb rich in phytoestrogens and a bioactive compounds, traditionally used to support female reproductive health [113–115]. Recent studies have investigated its potential effects on skeletal muscle function [116–118]. A RCT involving postmenopausal women demonstrated that six weeks of Shatavari supplementation (1000 mg/day) improved handgrip strength and increased myosin regulatory light chain phosphorylation in the vastus lateralis muscle, indicating enhanced myosin contractile function [117]. In the context of ST and *Shatavari* supplementation, one study was identified [68] (PEDro: 8). An 8-week supplementation regimen (1000 mg/day) did not induce significant increase in strength compared with ST alone, and effects on body composition and BMD were not evaluated. Consequently, no clear conclusions or recommendations can currently be made regarding these outcomes.

## Limitations

In addition to the important and novel findings of the review, there are also some limitations. Although a total of 34 studies were identified, the evidence regarding individual dietary strategies in combination with ST is severely limited. No studies on different dietary habits such as intermittent fasting, plant-based diet, or paleo diet in combination with ST could be identified. Similarly, only a few studies were conducted on the use of various dietary supplements such as amino acids, creatine, calcium or vitamin D in postmenopausal women with ST. Consequently, no clear recommendations or statements can be made. Furthermore, the study designs and training protocols of the identified studies are highly heterogeneous, making comparisons very challenging. In addition to the heterogeneous study designs, only a very limited number of studies have investigated the long-term effects of supplementation, with the majority of trials lasting no longer than 12 weeks. Furthermore, the definition of postmenopausal women was somewhat nonspecific across studies. Regarding study quality, most investigations achieved a PEDro score of 6 or higher, indicating a high methodological standard. Only the study by Molina et al. [57] and the two investigations by Ryan et al. [40, 41] scored below 6.

In addition, some studies included combined strength and endurance training along with various dietary strategies making it difficult to isolate the specific effects of strength training or dietary interventions. Consequently, these studies were not included in the review. Nevertheless combining strength and endurance exercise is essential for maintaining overall health and promoting longevity and is therefore equally recommended by the World Health Organization [119]. However, ST has been shown to be fundamentally important for maintaining or improving body composition, muscle strength and BMD. An exact dose-response relationship, however, cannot be determined, as this depends on a various factors such as training intensity, weekly volume, frequency, duration of the intervention and the level of supervision provided to participants.

## Conclusion

The present systematic review aimed to examine the effects of nutritional strategies in combination with ST on body composition, strength capacity and BMD in postmenopausal women. A total of 34 studies were identified. The evidence indicates that postmenopausal women should engage in structured and systematic ST. However, an exact dose-response relationship cannot be determined. For reducing BW and FM a CRD with a deficit of 250–750 kcal/day can be a beneficial strategy. A daily protein intake should be at least 0.8 g/kgBW/day to maintain LBM and muscle strength during CRD. The recommended amount can be attained through the consumption of high-protein foods, protein supplements or amino acids. The evidence on a high-protein diet (> 1.4 g/kgBW/day) in combination with ST in postmenopausal women is limited, making it impossible to draw definitive conclusions regarding potential additive or adverse effects. Regarding the use of dietary supplements in conjunction with ST, the limited number of available studies precludes drawing definitive conclusions. Consequently, the present findings and observations should be interpreted as exploratory. Therefore, further systematic investigations exploring diverse nutritional and supplementation strategies are required to generate more precise insights and develop evidence-based recommendations. A key consideration in all studies should be the implementation of structured and supervised training protocols to ensure sufficient training stimuli and accurately assessing potential additive effects.

## Supplementary Information

Below is the link to the electronic supplementary material.

Supplementary Material 1

Supplementary Material 2

## Data Availability

Data described in the manuscript, code book, and analytic code will be made available upon request pending.

## Abbreviations

AHA: American Heart Association
BCAA: Branched-chain amino acids
BF: Body fat
BF%: Body fat percentage
BMI: Body Mass Index
BMD: Bone mineral density
BRMR: Baseline resting metabolic rate
BW: Body Weight
CRD: Calorie-restrictive diets
DHA: Docosahexaenoic acid
EFSA: European Food Safety Association
EPA: Eicosapentaenoic acid
FFM: Fat-free mass
FM: Fat mass
FO: Omega-3-fatty acids
kgBW: Kilogram bodyweight
kgBW/d: Kilogram bodyweight per day
LCi: L-citrulline
LBM: Lean body mass
Mdiff: mean of the difference before and after the intervention
MeSH: Medical Subject Headings
P: Protein
PRISMA: Preferred Reporting Items for Systematic Reviews and Meta-Analysis
RCTs: Randomized, controlled study designs
SD: Standard deviation
SDdiff: Standard deviation of the difference before and after the intervention
SE: Standard error
SMM: Skeletal muscle mass
ST: Strength training
WC: Waist circumference
Z: Zataria multiflora
